# Cancer brain metastasis: molecular mechanisms and therapeutic strategies

**DOI:** 10.1186/s43556-025-00251-0

**Published:** 2025-02-25

**Authors:** Yu Lu, Yunhang Huang, Chenyan Zhu, Zhidan Li, Bin Zhang, Hui Sheng, Haotai Li, Xixi Liu, Zhongwen Xu, Yi Wen, Jing Zhang, Liguo Zhang

**Affiliations:** 1 Department of Biotherapy, Cancer Center and State Key Laboratory of Biotherapy, West China Hospital, Sichuan University, Chengdu, 610041 China; 2 Center for Translational Medicine, Key Laboratory of Birth Defects and Related Disease of Women and Children of MOE, West China Second University Hospital, Sichuan University, Chengdu, 610041 China; 3 Department of Neurosurgery, West China Hospital, Sichuan University, Chengdu, 610041 China

**Keywords:** Brain metastasis, Lung cancer, Therapy, Prognosis, Blood–brain barrier

## Abstract

Brain metastases (BMs) are the most common intracranial tumors in adults and the major cause of cancer-related morbidity and mortality. The occurrence of BMs varies according to the type of primary tumors with most frequence in lung cancer, melanoma and breast cancer. Among of them, lung cancer has been reported to have a higher risk of BMs than other types of cancers with 40 ~ 50% of such patients will develop BMs during the course of disease. BMs lead to many neurological complications and result in a poor quality of life and short life span. Although the treatment strategies were improved for brain tumors in the past decades, the prognosis of BMs patients is grim. Poorly understanding of the molecular and cellular characteristics of BMs and the complicated interaction with brain microenvironment are the major reasons for the dismal prognosis of BM patients. Recent studies have enhanced understanding of the mechanisms of BMs. The newly identified potential therapeutic targets and the advanced therapeutic strategies have brought light for a better cure of BMs. In this review, we summarized the mechanisms of BMs during the metastatic course, the molecular and cellular landscapes of BMs, and the advances of novel drug delivery systems for overcoming the obstruction of blood–brain barrier (BBB). We further discussed the challenges of the emerging therapeutic strategies, such as synergistic approach of combining targeted therapy with immunotherapy, which will provide vital clues for realizing the precise and personalized medicine for BM patients in the future.

## Introduction

Brain metastases (BMs) are the most common intracranial tumors, which exhibit more than ten times of incidence than that of primary brain tumors [1]. Around 20% of cancer patients will develop BMs with lung cancer is the most frequent tumor type that metastasizes to brain [2]. Almost half of lung cancer develop into BMs, followed by breast cancer (20%) and melanoma (15%) [3]. Lung cancer is divided into non-small cell lung cancer (NSCLC) and small cell lung cancer (SCLC) based on the histopathology [4, 5]. Among NSCLC patients, lung adenocarcinoma (LUAD, ~ 58.6%) and large cell carcinoma (~ 17.7%) carry a higher frequency for BMs compared to squamous cell carcinoma (~ 9.9%) [5]. For SCLC, approximately 10% of patients have BMs at the time of diagnosis and 40–50% will develop BMs during the course of disease [6, 7]. Lung cancer brain metastasis (LCBM) frequently occurs in patients with advanced stage and leads to poor overall survival (OS) [8, 9].

“Seed and soil” hypothesis posits that BMs are resulted from the interaction between disseminated cancer cells (DCCs) and brain microenvironment. Tumor cells disseminate from primary lung cancer, enter the blood circulation system and become the circulating tumor cells (CTCs). After spreading throughout the circulatory system to the brain microvasculature, CTCs extravasate the BBB and locate into brain, where it is a complex organ and favorable soil for DCCs. To successfully outgrow in brain, DCCs interact with brain resident cellular and acellular components to adapt the brain microenvironment. Brain resident cells include neurons, astrocytes, oligodendrocytes, immune cells and vascular-related cells. Of note, the blood–brain barrier (BBB), composed of endothelial cells, pericytes and astrocyte endfeets, presents a formidable structure for tumor cells to penetrate. Once formation of BMs, blood-tumor barrier (BTB) gradually replaces the BBB, exhibiting different permeability and characteristics [10]. The distinctive microenvironment of brain contains resident cell populations, structural composition, and immune processes [11, 12], which means that cancer cells seeding into the brain need possess the capacities to not only penetrate the BBB but also to survive and proliferate in the metastatic niche. Gain of unique driver mutations and metabolic reprogramming are critical for BM tumor cells to adapt the brain microenvironment. Current therapeutic approaches of BMs include surgical resection, radiotherapy, targeted therapy, immunotherapy or a combination of several modalities [13–15]. Although systemic therapies exhibit promising effects for BMs treatment, the impermeable property of BBB limits the transport of therapeutic agents to the brain, making systemic therapies less effective in treating BMs than in treating other types of cancers. Current biomaterial-based nanodrugs have been developed and applied in preclinical studies and clinical trials of cancer immunotherapies to overcome the obstruction of BBB.

BM patients exhibit significant neurological complications, resulting in a poor quality of life and short life span. Despite undergoing rough treatment, BM patients still exhibit very grim prognosis. Therefore, understanding the underlying mechanisms of BMs is an urgent need to develop effective therapeutic strategies. Recent studies have enhanced understanding of the mechanisms of BMs. The newly identified potential therapeutic targets and the advanced therapeutic strategies have brought light for a better cure of BMs. In this review, we comprehensively described the major steps of metastatic spreading of cancer cells to brain and addressed the influence of brain microenvironment and the molecular determinants of progression. Furthermore, we summarized the current treatment approaches and discussed the challenges of the emerging therapeutic strategies that may improve the prognosis of BM patients.

## Molecular mechanisms of brain metastases

Cancer BMs is complex and contains multiple steps, including epithelial-mesenchymal transition (EMT), detachment from the primary lung cancer, intravasation of blood vessels, hematogenous dissemination, penetrating the BBB, and finally adaption of brain microenvironment and colonization in the brain niche. Each step of BM is tightly regulated by crucial molecular and cellular mechanisms.

### Tumor cell intravasation and extravasation

#### The role of EMT for brain metastases initiation and intravasation

Detachment from primary tumor and intravasation of blood vessels is the first step of distal metastasis of cancer cells to the brain. The process is characterized by gain of EMT feature for cancer cells to break through the basement membrane at the primary tumor sites [16–18]. Primary tumor cells are immotile and tightly bound to each other and to the neighboring extracellular matrix (ECM). EMT is the trans-differentiation process through which primary tumor cells can be transformed to develop the ability to detach from primary tumor and invade into surrounding tissues. EMT can drive tumor cell to gain a mesenchymal phenotype, 13 which is crucial for tumor progression and metastasis [19].

EMT in primary tumor cells shifts between different intermediate stages with different invasive, metastatic, and differentiation characteristics [20]. These various stages possess diverse cellular characteristics, chromatin landscapes, and gene expression profiles that are regulated by distinct transcription factors and signaling pathways. EMT can be induced by activation of various growth factor receptor tyrosine kinases and cellular signaling pathways, including the TGF-β, Wnt and Notch pathways [21, 22]. TWIST and SNAIL, the markers of EMT, are overexpressed in LCBM tumors [8]. During the EMT process, LCBM cells exhibit high expression level of CD44 and stem cell-like properties. Driver mutations have been reported to enhance EMT transformation and contribute to the LCBM. EMT is often driven by transcription factors, which trend to repress epithelial genes and activate mesenchymal genes [23]. Epigenetic and post-translational modulators also play a vital role in controlling the EMT process. Moreover, stromal cells within tumor microenvironments (TME) can induce EMT of tumor cells. It has been reported that metastatic tumor cells with mesenchymal phenotype usually proliferate near endothelial and inflammatory cells. Metastasis tumor cells release chemokines to attract immune cells and stimulate angiogenesis, thus promoting the development of a unique inflammatory and highly vascularized niche [20]. Cancer-associated fibroblasts (CAFs) have been reported to enhance EMT and drive primary tumor cells migration [24]. In addition, other TME factors, such as hypoxia, metabolic stressors, and matrix stiffness also could trigger the EMT process in primary tumor cells [25, 26].

#### Interactions with the vasculature and the blood–brain barrier

**Fig. 1:**
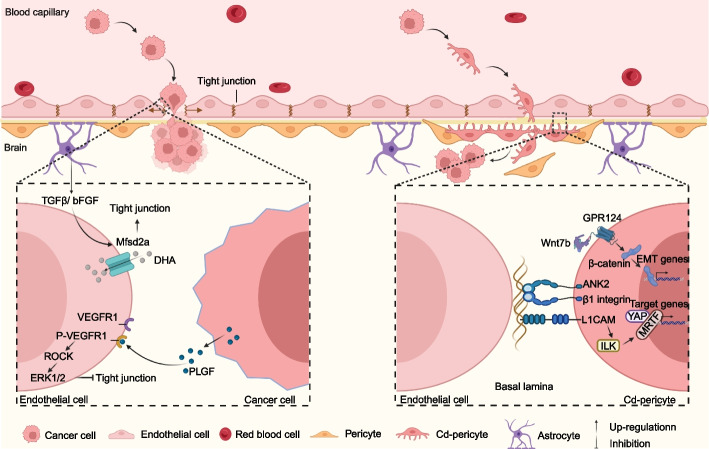
Molecular mechanisms of CTCs to penetrate the BBB during BM. Cancer cells expressed PLGF can active VEGFR1 and the downstream ROCK-ERK1/2 pathway and leads to the disassembly of the tight junction. Pathologically decrease of astrocyte-derived TGFβ/bFGF can also disassemble the tight junction by downregulation of Nfsd2a. In additiona, lung cancer cell-derived Cd-pericytes enhance BM formation through GPR124-mediated TEM and L1CAM-YAP/MRTF pathway-mediated spreading along brain capillaries. The figure was generated using Biorender.com

The BBB is a tight barrier structure between blood and brain tissue, which ensures the stability of brain microenvironment. BBB is composed of endothelial cells, pericytes and astrocyte endfeets and functions as a highly selective semipermeable border in brain to avoid the damage of biological agents and other risk factors [27]. BBB destruction by tumor cells is a key step during the formation of BM, but the cellular and molecular mechanisms involved in lung cancer cells penetrating BBB have not been fully understood. BBB is the first obstacle for metastatic cancer cells to colonize in brain and limited information about CTCs extravasating BBB has been demonstrated (Fig. 1). Due to the brain tumor burden, the tight junctions of BBB should be extended and the permeability of BBB increases at the site where CTCs extravasate into brain [28].

Disruption of BBB by cancer cells is linked to BM initiation and progression. However, the mechanisms of driving these events remain poorly understood. Li et al. have uncovered that SCLC patients with high levels of placental growth factor (PLGF) were prone to BM by promoting SCLC cell trans-endothelial migration (TEM) [29]. PLGF derived from SCLC cells triggers VEGFR1-ROCK-ERK1/2 axis activation and leads to disassembly of tight junctions in BBB. Downregulation of PLGF suppresses SCLC cell metastasis to brain in an experimental BM model. Tiwary et al. have demonstrated that BBB was disrupted in BM by downregulation of major facilitator superfamily domain 2a (Mfsd2a), the endothelial cell-expressed docosahexaenoic acid (DHA) transporter [30]. Pathologically diminished TGFβ and bFGF pathways in endothelium cells leads to downregulation of Mfsd2a accompanied with reduced DHA transport and altered lipid metabolism, suggesting that restoring DHA metabolism may be a novel therapeutic strategy to block BM. Previous studies revealed that breast cancer cells dysregulated the action dynamics of brain endothelial cells and destructed BBB by releasing miRNA-181c-containing extracellular vesicles to facilitated BM of breast cancer [31]. Whether the same mechanism occurs in lung cancer cells is needed to be further verified. It has been reported that dynamic extravasation of CTCs in the small holes of brain vascular wall was essential for LCBM [32]. Another way of CTCs passing through the BBB may be by disrupting the endothelium to gain entry [33]. Two studies performed by Er et al. and Huang et al. have highlighted the importance of pericyte-like spreading during extravasation and co-option with brain capillaries. Er et al. indicated that mechano-transduction signaling triggered by the pericyte-like spreading of DCCs on brain capillaries was critical for metastatic colonization [34]. DCCs employed L1 cell adhesion molecule (L1CAM) to spread on capillaries and displaced resident pericytes. In addition, L1CAM activated mechano-transduction effectors YAP and MRTF by engaging β1 integrin and integrin-linked kinase to enhance the outgrowth of BM initiating cells both immediately following infiltration and after exiting from a period of latency. Huang et al. revealed that CD44^+^ lung cancer stem cell-derived pericyte-like cells (Cd-pericytes) enhanced BM formation through GPR124-mediated TEM [35]. Cd-pericytes exhibit remarkable TEM capacity to effectively intravasate into the vessel lumina, survive in the circulation, extravasate into the brain parenchyma, and then de-differentiate into cancer stem cells to form BM. Cd-pericytes expressed GPR124 mediates the activation of Wnt7b-catenin signaling to enhance TEM capacity for intravasation and extravasation, two critical steps during LCBM. Furthermore, selective disruption of Cd-pericytes, GPR124, or the Wnt7b-catenin signaling markedly reduced LCBM. Xia et al. have uncovered that Mesothelin (MSLN) expression was significantly elevated in both serum and tumor tissue from NSCLC-BM patients and was correlated with a poor clinical prognosis [36]. MSLN significantly enhanced the BM abilities of NSCLC cells by facilitating the expression and activation of MET through the c-Jun N-terminal kinase signaling pathway, which allowed tumor cells to disrupt tight junctions and the integrity of the BBB. Drugs targeting MSLN and MET effectively blocked the development of BM and prolonged the survival in mice model.

### Survival and colonization of circulating tumour cells in brain

#### Survival of circulating tumour cells

Tumor progression is often accompanied by new blood vessel formation, which allows tumor cells to access the blood circulation and long-distance metastasis [37]. Tumor cells at the primary site acquire invasive properties, break away from the primary tumor, and intravasate to blood vessels to become CTCs. Blood vessel density is one of the most import factors for lung cancer intravasation [38]. Donnem et al. revealed that tumor cells hijacked the host vasculature to obtain blood supply and migrate to distinct locations along the vessels. Approximately 20% of NSCLC patients exhibit vessel co-option [39]. Upon entering the circulation, CTCs derived from primary lung cancer are exposed to tremendous physical and biochemical stresses, such as high oxygen tension, fluid shear stress, immune surveillance and apoptosis. These stresses lead to extreme oxidative stress characterized by increased reactive oxygen species (ROS) in CTCs, hindering the survival of the vast majority of CTCs.

**Fig. 2:**
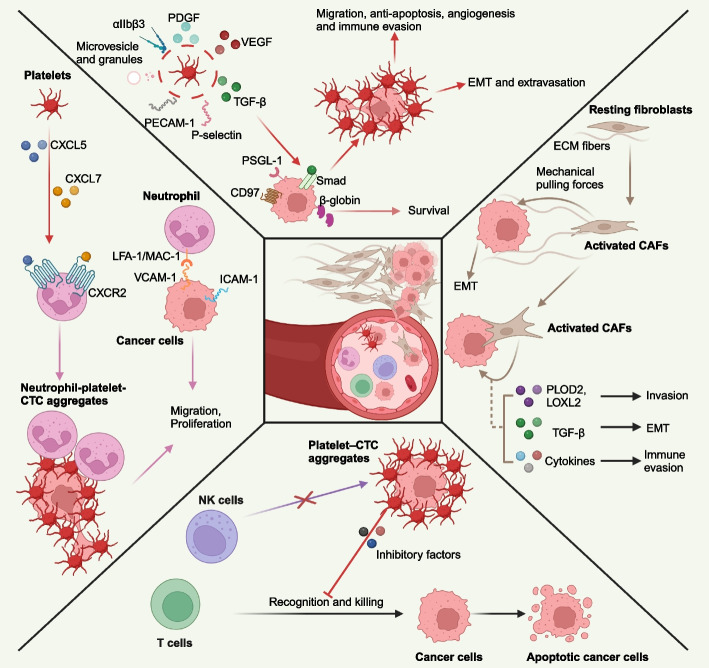
The interactions between CTCs and the blood cells, with a focus on the main molecules involved in these interactions. Platelets derived TGF-β binds to Smad of CTCs to promote the invasion of CTCs. The expression of PECAM-1, P-selectin, VEGF, PDGF and αIIbβ3 integrin, as well as the release of microvesicle and granules are furthermore beneficial for the migration, anti-apoptosis, angiogenesis and immune evasion of CTCs. CTCs can express β-globin to promote itself survival. CTCs derived adhesion molecules VCAM-1 and ICAM-1 bind to LFA-1/MAC-1 receptors on neutrophils to promote the migration of the CTCs. CXCL5/7-CXCR2 signaling enhances the formation of neutrophils-platelets-CTCs aggregates to promote the proliferation of CTCs. CAFs can promote CTCs migration through mechanical pulling forces on ECM fibers and facilitate invasion and immune evasion of CTCs through releasing PLOD2, LOXL2, TGF-β and other cytokines. Platelets-CTCs aggregates can protect CTCs from recognition by NK cells and T cells. The figure was generated using Biorender.com

Although current advances in enriching and analyzing rare cells in the blood stream have allowed to further characterize CTCs [40], the mechanism by which CTCs escape immune surveillance and survive in the circulation remain poorly understood (Fig. 2). By performing single-cell RNA-seq (scRNA-seq) of CTCs from lung cancer patients, Zheng et al. have revealed that increased intracellular ROS in CTCs could consistently induct the production of β-globin, which in turn efficiently suppresses the level of ROS and mediates the survival of CTCs [41]. CTCs develop strategies, such as interacting with neutrophils, platelets and CAFs to escape immune surveillance in the blood vascular system. CTCs can also travel as clusters to provide coagulation and adherence to endothelial cells, resulting in reducing the speed of blood flow, facilitating the roll-over on endothelial cells and finally extravasating from the circulation. Duda et al. have uncovered that BMs from LUAD and other carcinomas in patients contained CAFs in contrast to primary brain tumors or normal brain tissue [42]. Fibroblasts carried over from the primary tumor increase the efficiency of lung metastasis. Ao et al. have reported that circulated CAFs (cCAFs) were found in the peripheral blood of breast cancer patients with metastatic disease using microfilter technology [43]. The presence of cCAFs was associated with clinical metastasis, indicating that cCAFs may complement CTCs as a clinically relevant biomarker for metastatic cancers. On the other hand, CAFs can generate gaps in the basement membrane to promote CTCs migration by applying mechanical pulling forces on ECM fibers [44]. In addition, CAFs derived collagen modifying enzymes, such as PLOD2 and LOXL2, can mediate ECM remodeling and stiffness to enhance CTCs invasion and metastasis [45]. Furthermore, CAFs can protect CTCs from antitumor immune response by secretion of TGF-β and interleukin 6 (IL-6) [46, 47].

In the circulation, CTCs are no longer protected by the primary TME and are more exposed to immune cells, including circulating T lymphocytes and natural killer (NK) cells [48]. CTCs can interact with and co-opt other circulating cell types to evade the immune system and drive metastasis. Recent studies have provided insights into the mechanism of the increased metastatic potential mediated by platelets. The platelets play a vital role in promoting survival, escape from immune surveillance in the bloodstream, and ultimately support metastasis of CTCs [49, 50]. The platelets can release adhesion molecules and growth factors, including integrins (αIIbβ3), selectins (P-selectin), immunoglobulin superfamily proteins (PECAM-1), platelet-derived growth factor (PDGF), and vascular endothelial growth factor (VEGF), to form platelet-CTC aggregates to promote the anti-apoptosis, angiogenesis and immune evasion of CTCs [51, 52]. In addition, platelets exhibit anti-apoptosis behaviors of CTCs by secreting platelet microvesicles, or releasing of platelet granules [53]. Based on NanoString analysis of CTCs RNAs and plasma cell-free RNAs, Beck et al. have identified platelet-associated genes, such as platelet factor 4*, ﻿CLU*, serglycin secreted protein acidic and rich in cysteine *and* serglycin, in LCBM that could predict OS in LCBM patients, suggesting an important role that platelets play in LCBM [54]. The function of these platelet-associated genes for LCBM still need to be confirmed in the future. Furthermore, platelet-derived TGF-β can activate the TGFβ-Smad and NF-κB pathways in cancer cells, resulting in their transition to an invasive mesenchymal-like phenotype and enhanced metastasis [50]. Platelets also can recruit neutrophils by CXCL5/7-CXCR2 signaling to form neutrophil-platelet-CTC aggregates to enhance early metastatic niches formation and significantly enhance metastatic seeding and progression [49].

Szczerba et al. have isolated and characterized CTCs associated white blood cells (WBCs), as well as corresponding cancer cells within each CTCs-WBCs cluster from patients with breast cancer [55]. By performing scRNA-seq, they revealed that CTCs were associated with neutrophils in the majority of these cases. In addition, a number of cell cycle progression associated genes were highly expressed in CTCs associated with neutrophils against those of CTCs alone. Furthermore, cell–cell junctions and cytokine-receptor pairs that define CTCs-neutrophils clusters were identified, representing key vulnerabilities of the metastatic process. Thus, the association between neutrophils and CTCs drives cell cycle progression within the bloodstream and expands the metastatic potential of CTCs, providing a rationale for targeting this interaction in treatment of LCBM.

#### Metabolic reprogramming and colonization in brain

The BM cells depend not only on the extravasation of blood vessels, but also on the post metastatic survival and colonization in brain. Once lung cancer cells extravasate and settle in brain, they must overcome the impact of brain microenvironment. While normal brain cells depend on glucose for energy production, BM cells in brain undergo metabolic reprogramming and depend not only on glucose, but also on glutamine for energy [56]. These metabolic adaptations are the result of interactions between BM cells and brain cells including astrocytes and neurons, which promote rapid metastatic colonization in brain [57, 58].

Recent studies have revealed that metabolic reprogramming enhanced the adaption of brain microenvironment and colonization of BM cells. By performing multi‑omic molecular profiling, Fukumura et al. have demonstrated that enhanced oxidative phosphorylation (OXPHOS) was found in LCBM and other BM types compared to patient-matched primary or extracranial metastatic tissues, which might be resulted from PI3K-AKT pathway activation [59]. Ngo et al. have highlighted metabolic constraints imposed by the serine-limited brain environment restricted metastatic tumor growth [60]. 3-phosphoglycerate dehydrogenase (PHGDH), a catalyst in the serine synthesis pathway, was identified as a major determinant of BM for NSCLC and other cancer types. Genetic suppression or pharmacologic inhibition of PHGDH attenuated BM rather than extracranial tumor growth. Zeng et al. have uncovered that BM breast cancer cells expressed N-methyl-d-aspartate receptors (NMDARs) and utilized the glutamate secreted by glutamatergic neurons to promote metastatic colonization in the brain [61]. BM cells can form pseudo-tripartite synapses with glutamatergic neurons to obtain the glutamate. Autocrine secretion of glutamate by neurons stimulates NMDARs on the member of BM cells, which is transduced via the cytoplasmic adaptor protein guanylate kinase-associated protein to enhance the outgrowth and colonization. Mitochondrial metabolism can generate energy, regulate redox homeostasis, and provide key metabolites for macromolecule synthesis. Recently, targeting mitochondrial metabolism has emerged as a treatment option for LCBM patients. Luttman et al. revealed that abelson (ABL) tyrosine kinases promoted lung cancer cells metastases and the ABL kinase allosteric inhibitors could impair mitochondrial integrity, decrease OXPHOS and lead to apoptosis of LCBM cells [62]. By utilizing a clustered regularly interspaced short palindromic repeats loss-of-function screen, they further identified HMG-CoA reductase, the target of statin therapies, as a top-scoring sensitizer to ABL inhibition. Metabolic combination treatment with ABL allosteric inhibitors and statins decreases LCBM cells survival in a synergistic manner. Cheng et al. have modified the antiglycolytic drug lonidamine (LND) to mitochondria-targeted mito-lonidamine (Mito-LND), which showed 100-fold more potent [63]. The Mito-LND exhibits a tumor-selective inhibition of OXPHOS and inhibits mitochondrial bioenergetics in lung cancer cells, leads to reduced LCBM in mice model. In summary, metabolism targeted therapy maybe effective to decrease metastatic outgrowth, leading to increased survival for LCBM patients.

#### Dormancy in brain

**Fig. 3:**
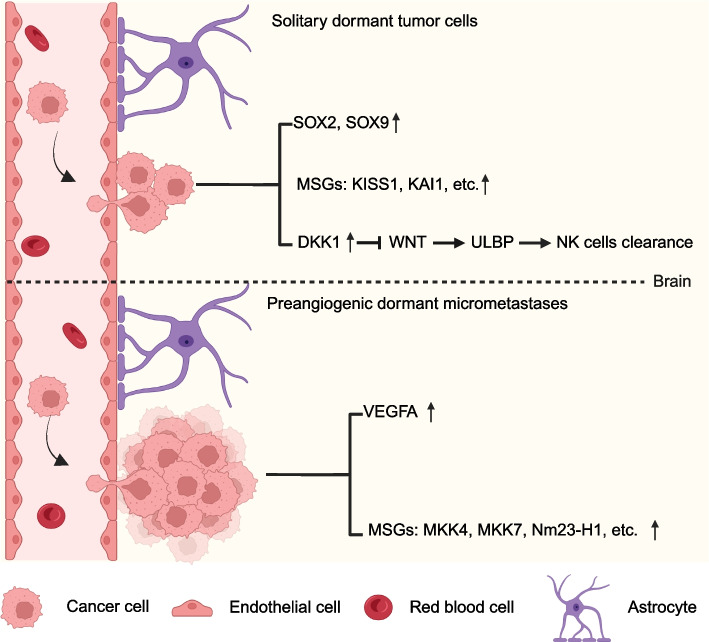
The dormant patterns of DCCs in the site of LCBM. There are two types of metastatic dormancy that help DCCs to avoid apoptosis and immune surveillance. Solidary dormant tumor cells exhibit stem cell signature with upregulation of SOX2, SOX9, KISS1, KAI1, and avoid NK cell clearance by DKK1-mediated WNT pathway inhibition. Preangiogenic dormant micrometastases are characterized by upregulated expression of VEGFA, MKK4, MKK7 AND Nm23-H1. The figure was generated using Biorender.com

Once extravasating BBB and settling in brain, most lung cancer cells die or enter a dormant state. Dormant lung cancer cells can remain unaffected by standard chemotherapy and may develop to tumor burden years after surgical resection. Dormancy may be the result of an adaptive response that allows DCCs to pause the proliferation and avoid stress imposed by microenvironment or treatment. There are two different types of metastatic dormancy, including solitary dormant tumor cells and preangiogenic dormant micrometastases (Fig. 3). Both these two kinds of dormant cells can remain dormant for months or years, leading to uncertainty in the prognosis for BM patients. The solitary dormant tumor cells spread the distant site with the characteristics of neither proliferation nor apoptosis. While the preangiogenic dormant micrometastases function as a potential contributor to metastatic dormancy of the small tumor cells clusters with a balanced rate of cell proliferation and death [64].

Different mechanisms have been reported to responsible for metastatic dormancy. Malladi et al. have demonstrated that dormant lung cancer cells expressed SOX2 and SOX9 transcription factors and imparted themselves the cancer stem cell identity, which was essential for their survival under immune surveillance and for metastatic outgrowth [65]. Through expression of the WNT inhibitor DKK1, these dormant cells exhibit a slow-cycling state with downregulation of UL16-binding proteins (ULBP) to evade the NK-cell-mediated clearance. Thus, once acquiring stem-like state and silencing WNT signaling, metastatic lung cancer cells can enter quiescence and evade innate immunity to remain latent for extended periods. By using multiphoton laser scanning technology, Kienast et al. imaged the single step of metastasis formation in real time and revealed that long­term dormant cells were all solitary or in tiny clusters of up to three cells in strict perivascular localization. Furthermore, early angiogenesis is essential for lung cancer cell metastasis and vascular endothelial growth factor A (VEGFA) inhibition induces long-term dormancy of lung cancer micrometastases by preventing angiogenic growth [32]. Horak et al. revealed that several metastasis suppressor genes, such as *KISS1, KAI1, MKK4/7* and *Nm23-H1* could induce dormancy of cancer cells during metastases [66]. KISS1 triggers dormancy in solitary metastatic tumor cells by causing growth arrest at the secondary site. KAI1 induces growth arrest prior to extravasation by binding a vascular endothelial cell surface marker. MKK4, MKK7 and Nm23-H1 appear to promote dormancy of micrometastatic colonies, after disseminated tumor cells have undergone several rounds of proliferation. In summary, dormancy should be a result of an adaptive response that allows DCCs to pause the proliferation and avoid stress imposed by microenvironment or treatment.

### Interactions with brain microenvironment

#### Crosstalk with astrocytes

**Fig. 4:**
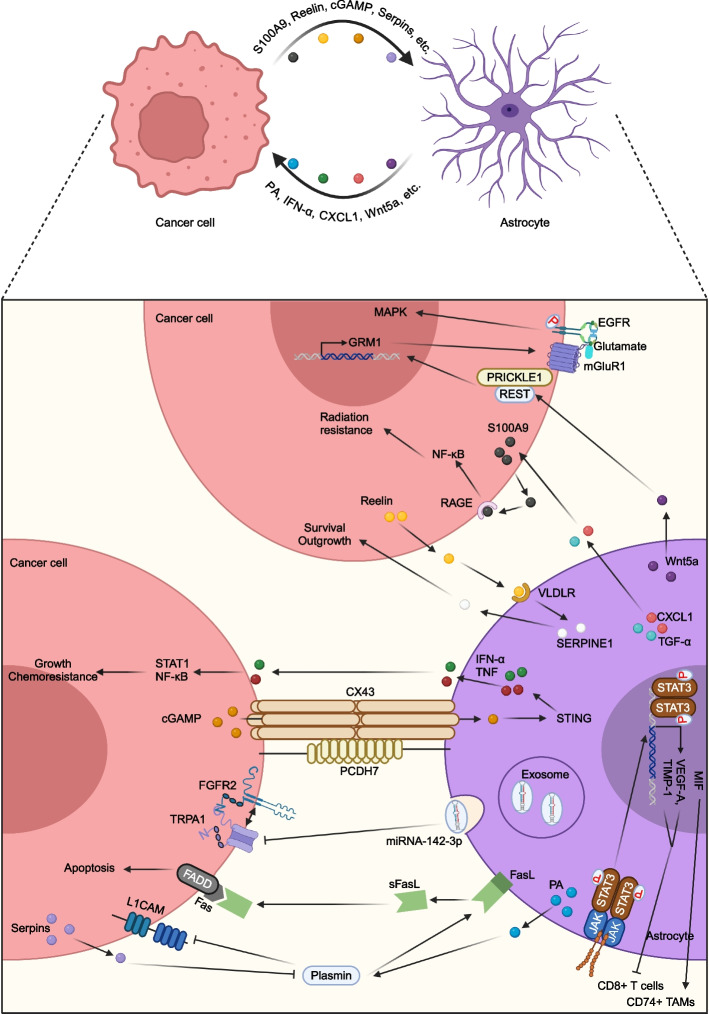
Crosstalk between lung cancer cells and astrocytes in the BM site. Astrocytes secrete PA to generate plasmin to promote cancer cell apoptosis through secreting sFasL and inhibiting L1CAM function. Whereas cancer cells express high level of anti-PA serpins to prevent above process. Astrocytes transfer the *TRPA1*-targeting exosomal miRNA-142-3p to deplete *TRPA1* in LCBM cells and in turn inhibit TRPA1-mediated activation of FGFR2 to hinder the metastatic process. Cancer cells express PCDH7 to promote the assembly of carcinoma-astrocyte gap junctions to transfer cGAMP to astrocytes, which in turn activates STING pathway in astrocytes and then produce cytokines to support LCBM. STAT3^+^ reactive astrocytes produce cytokines to generate immune suppressive TME to enhance LCBM. Cancer cells secrete Reelin to recruit reactive astrocytes to the TME and in turn astrocytes release SERPINE1 to promote cancer cells survival and outgrowth. Astrocytes secreted CXCL1 and TGF-α can induce S100A9 production in cancer cells to promote radiation resistance. Astrocytes derived Wnt5a induces mGluR1 expression in cancer cells, which directly interacts with and stabilizes EGFR to activate the downstream MAPK pathway to promote BM progression. The figure was generated using Biorender.com

When DCCs reach brain, they encounter a complex microenvironment that is different from the primary site. Since the brain microenvironment is a vital aspect that affect the progression of BM, dissecting the mechanisms of the interactions between DCCs and different components of the brain microenvironment will facilitate the understanding of biological behavior of BM. The brain microenvironment consists of specialized cell types, such as neuron, microglia, oligodendrocyte, astrocyte, and endothelial cell. Among them, astrocyte is the most abundant parenchymal cell type and possesses the biological function of supporting and maintenance of brain homeostasis. However, in a state of metastases, astrocytes are reactivated and interact with extravasated tumor cells to reconstruct a BM microenvironment (Fig. 4).

As the first brain cellular component that contact with lung cancer cells after extravasation, astrocytes play an important role for the formation of LCBM. Valiente et al. have illustrated that reactive astrocytes could release plasminogen activator (PA) in the presence of extravasated BM cells [67]. PA generates plasmin from neuron-derived plasminogen to mobilize FasL from astrocytes to kill extravasated BM cells. In addition, astrocytes produced plasmin can cleave and inactivate BM cells secreted L1CAM to prevent BM cells from spreading along brain capillaries and metastatic outgrowth. On the other hand, LCBM cells express high levels of anti-PA serpins to prevent plasmin generation and the metastasis-suppressive effects. Berrout et al. revealed that upon metastasis to the brain, astrocytes could transfer the *TRPA1*-targeting exosomal miRNA-142-3p to deplete *TRPA1* in LCBM cells and in turn inhibit TRPA1-mediated activation of FGFR2 to hinder the metastatic process [68]. Chen et al. demonstrated that astrocytes could promote LCBM by forming carcinoma-astrocyte gap junctions [69]. LCBM cells express PCDH7 to promote the assembly of carcinoma-astrocyte gap junctions and use these channels to transfer 2′3′-cyclic GMP-AMP (cGAMP) to astrocytes, which in turn activating the stimulator of interferon genes (STING) pathway in astrocytes. Activated astrocytes then product cytokines such as IFN-α and TNF to active STAT1 and NF-κB pathways in lung cancer cells, thereby supporting LCBM tumor growth and chemoresistance. Priego et al. revealed that a subpopulation of STAT3^+^ reactive astrocytes were required for LCBM by both promoting the expansion of CD74^+^ tumor associated macrophages (TAMs) and negatively regulating the activation of CD8^+^ T cells, which could produce immunosuppressive microenvironment to enhance LCBM [70]. Monteiro et al. have identified the astrocytes-released cytokines such as CXCL1 and TGF-α that could induce S100A9 secretion in LCBM cells [71]. The activation of the S100A9-RAGE-NF-κB-JUNB pathway in LCBM cells functions as a potential mediator for radiotherapy-resistance. Inhibition of this pathway can increase the therapeutic benefits in vivo at lower doses of radiation. Qu et al. have demonstrated that SCLC cells could secrete Reelin to recruit reactive astrocytes to the TME [72]. Reactivated astrocytes in turn secrete neuronal pro-survival factors such as SERPINE1 to promote SCLC cells survival and outgrowth. Ishibashi et al. revealed that astrocytes-derived Wnt5a induced mGluR1 expression in LCBM cells, which directly interacted with and stabilized EGFR in a glutamate-dependent manner, leading to activation of the mitogen-activated protein kinase (MAPK) pathway and progression of LCBM [73]. Collectively, reciprocal interactions between LCBM cells and astrocytes facilitate the adaption of lung cancer cells to brain microenvironment and acquire the therapeutic resistance.

#### Modulation by immune microenvironment

Brain is generally considered as an immune-privileged organ for DCCs to escape systemic chemotherapy and immunotherapy. To successfully outgrow in the brain, DCCs must overcome immune surveillance by interacting with both brain resident and infiltrated immune cells. Microglia, the brain resident macrophages, is the major immune cell type in brain that modulates the DCCs to adapt the brain immune microenvironment. The infiltrated bone-marrow-derived myeloid cells (BMDMs) cooperate with microglia to regulate brain immunity for LCBM. Lower programmed death ligand 1 (PD-L1) expression and less CD8^+^ T-cell infiltration was found in LCBM tumors compared with matched primary lung tumors, suggesting an immunosuppressive microenvironment for LCBM.

**Fig. 5:**
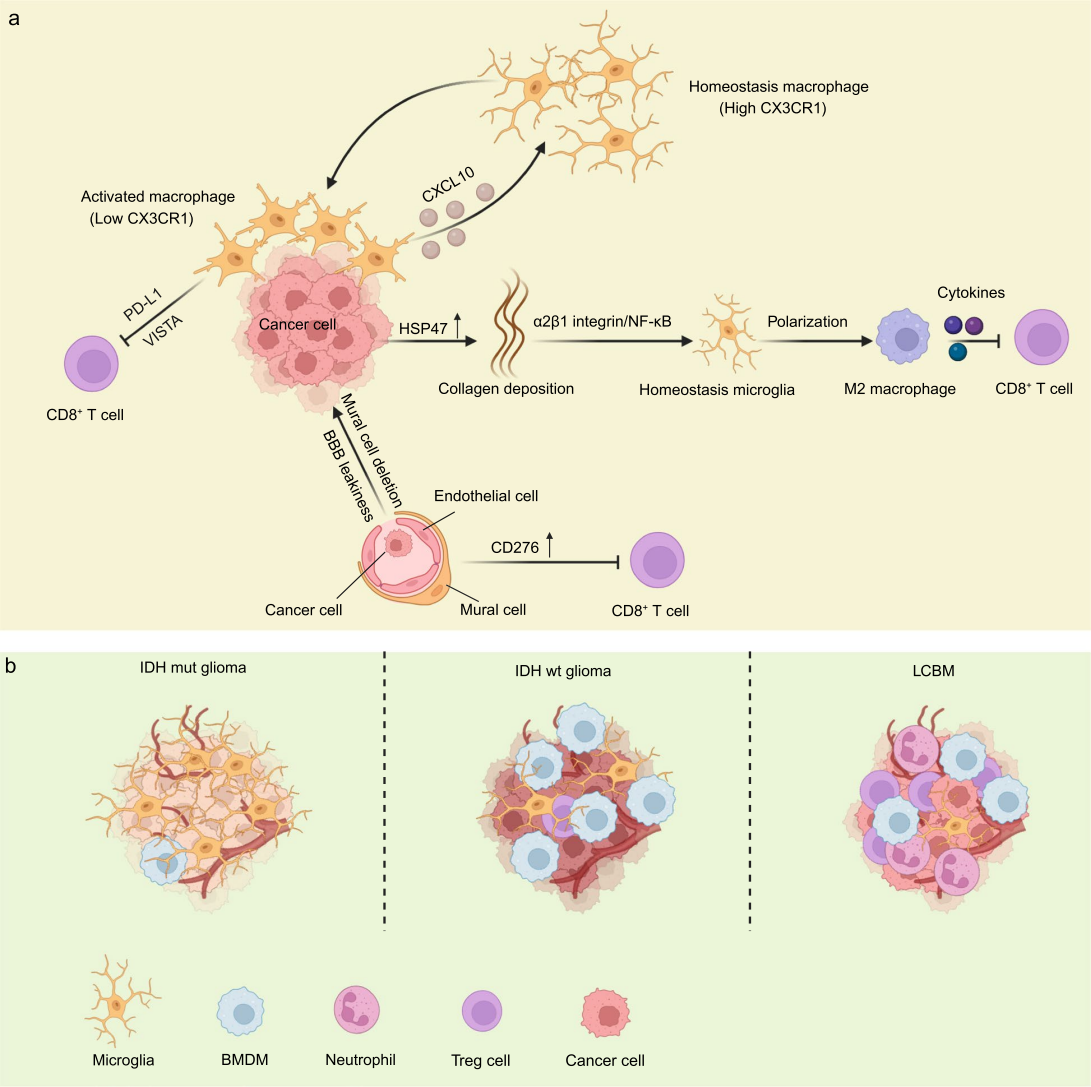
Interactions between lung cancer cells and immune cells in BM microenvironment. **a** Downregulation of CXCR1 in brain homeostasis macrophages lead to CXCL10 secretion and recruitment of VISTA^Hi^PD-L1^+^ immunosuppressive macrophages to BM lesions, which inhibit cytotoxic T cells and promote BM outgrowth. Upregulation of HSP47 in cancer cells can mediate collagen deposition, which in turn promotes macrophages polarization to M2 phenotype and represses CD8^+^ T cell mediated anti-tumour responses. Highly expressed CD276 in endothelial cells and mural cells of BM vasculature can lead to increased BBB leakiness and decreased CD8^+^ T cell infiltration. **b** The main immune cells in the TME of LCBM and glioma. The figure was generated using Biorender.com

The heterogeneity and specific roles of immune cells in shaping brain niche to regulate LCBM cells outgrowth were uncovered recently (Fig. 5a). Guldner et al. have demonstrated that brain resident microglia rather than BMDMs promoted BM outgrowth [74]. CX3CR1 downregulation in microglia led to an enriched interferon response signature and CXCL10 upregulation, which in turn recruited VISTA^Hi^PD-L1^+^ immunosuppressive microglia to BM lesions and finally promoted BM outgrowth. Correspondingly, inhibiting VISTA and PD-L1 signaling relieved immune suppression and reduced BM burden. Bejarano et al. have performed single-cell and bulk RNA-seq of vascular cells from human LCBM and healthy brain respectively. CD276 was identified as a potential immunotherapeutic target for BM [75]. Upregulation of CD276 in the BM vasculature can inhibit the infiltration of cytotoxic T cells. Anti-CD276 blocking antibodies treatment increased infiltrating CD8^+^ T cells in BM lesions and prolonged survival. Wang et al. revealed that upregulation of HSP47 in primary lung cancer cells could drive metastatic colonization and outgrowth in brain by creating an immunosuppressive microenvironment [76]. HSP47-mediated collagen deposition in the metastatic niche promotes microglia polarization to the M2 phenotype via the α2β1 integrin/NF-kB pathway, which upregulates the anti-inflammatory cytokines and represses CD8^+^ T cell mediated anti-tumor responses. Blocking the HSP47-collagen axis represents a promising therapeutic strategy against BM.

## Molecular and cellular landscapes of lung cancer brain metastasis

### Genetic and molecular drivers of lung cancer brain metastasis

Activation of epidermal growth factor receptor (EGFR) or anaplastic-lymphoma-kinase (ALK) is particularly important for lung cancer cells to survive and outgrowth in brain [3]. Inhibitors for EGFR and ALK have been used for targetable treatment in LCBM therapeutic practice (Table 1) [3, 11]. EGFR mutant NSCLC patients have longer OS and more likely to develop BM than patients with wild type EGFR [77]. Since EGFR mutant NSCLC patients had a higher incidence of BM at the time of diagnosis, an inherent propensity for BM should be exist in EGFR mutant NSCLC patients [78]. Shih et al. have performed whole-exome sequencing (WES) of 73 LCBM specimens and compared the mutations with that of primary lung cancers. Amplification of *MYC, YAP1, MMP3* and deletion of *CDKN2A/B* were identified among the highest frequency of driver mutations for BM [79]. Enhanced LCBM by overexpression of *MYC, YAP1 or MMP13* was confirmed in xenograft mouse models. Skakodub et al. have reported a cohort of 233 patients with sequenced NSCLC-BM cases and analyzed metastatic specimens with matched primary tumors. *﻿CDKN2A/B* alterations and cell cycle pathway alterations were found more common in the BM samples compared to primary tumors [80]. Alterations for genes of *TP53, MYC, SMARCA4, RB1, ARID1A,* and *FOXA1*, also exhibited significant enrichment in primary tumor specimens from patients who developed BM compared to those who did not have BM. Other driver mutations, such as ROS proto-oncogene 1 (*ROS1*) rearrangement, amplification or exon 14 mutations of mesenchymal-to-epithelial transition (*MET*), Neurotrophic tropomyosin receptor kinase (*NTRK*) fusion, and rearranged during transfection (*RET*) fusion were also identified in LCBM patients. Only a few studies have been reported for molecular characteristics of SCLC-BM patients so far. c-Kit overexpression, EGFR mutation, VEGF overexpression, PI3K activation, PTEN mutation, and Myc activation are among the most frequent molecular drivers for SCLC-BM [81]. These molecular abnormalities represent potentially actionable targets for drug development to treat aggressive LCBM.

**Table 1:** Driver mutations in patients with lung cancer brain metastasis

Driver mutations	Incidence of BM (%)	Mutation types	Encoded Protein	Function	Targeted drugs
*EGFR*	40 [82] 50.3 [83] ﻿23 ~ 32 [84] 44 [85]	Exon 19 deletion or point mutation in exon 21	Epidermal growth factor receptor tyrosine kinase	Regulate cell proliferation, migration, survival and differentiation	Erlotinib; Gefitinib; Rociletinib, Osimertinib and Zorifertinib
*ALK*	20 ~ 40 [84] 39.1 [86] 35 ~ 50 [87]	Chromosomal rearrangement	A member of the insulin receptor protein-tyrosine kinase superfamily	Regulate brain development and specific neurons formation in CNS	Crizotinib; Alectinib, Ceritinib; Brigatinib; Lorlatinib; Ropotrectinib
*KRAS*	32 [80] 6.7 [82]	Fusion	A member of small GTPase superfamily	Regulate cell proliferation	Sotorasib, Adagrasib
*ROS1*	19 [84] 19.4 [86]	Chromosomal rearrangement	A type I integral membrane protein with tyrosine kinase activity	Promote cell proliferation and differentiation	Crizotinib; Lorlatinib; Entrectinib, Ropotrectinib
*TP53*	66.7 [82] 41 [83]	Missense mutation	A tumor suppressor protein	Induce cell cycle arrest, apoptosis, senescence and DNA repair	NA
*RET*	46 [84] 25 [88]	Fusion or rearrangement	A transmembrane receptor and member of the tyrosine protein kinase family	Promote cell differentiation, migration, survival and the development of CNS	Pralsetinib, selpercatinib; Cabozantinib, Vandetanib and Sunitinib
*MET*	20 [84]	Genomic amplification or exon 14 skipping mutation	The receptor tyrosine kinase family	Regulates cell survival, migration and invasion	Capmatinib; Tepotinib, Savolitinib
*MYC*	12 ~ 21 [79]	Amplification or rearrangement	A proto-oncogene that encodes a transcription factor	Regulate cell cycle progression and cellular transformation	NA
*YAP1*	7 ~ 9 [79]	Amplification	A downstream nuclear effector of the Hippo signaling pathway	Regulate cell cycle, proliferation, EMT process and metastasis	NA
*MMP13*	9 ~ 10 [79]	Somatic copy-number alteration	A member of matrix metalloproteinase family	Promote the degradation of extracellular matrix proteins	NA
*CDKN2A/B*	27 [79] 34 [80]	Copy number loss	The cyclin-dependent kinase inhibitor 2A/B	Inhibit cell cycle progression	NA
*NTRK*	22 [84]	Fusion	Neurotrophic tropomyosin receptor kinase	Regulate the MAPK, PI3K, and PKC pathways	Larotrectinib; Ropotrectinib

*NA* not applicable

### Role of non-coding RNAs in lung cancer brain metastasis

Besides the driver mutations of protein-coding genes, non-coding RNAs (ncRNAs), which are essential in regulating pre-and post-transcriptional steps in protein synthesis [89–91], were also considered to play important roles for LCBM. NcRNAs play potential roles in determining BMs prognosis, chemo- and radio-resistance and may work as potential therapeutic targets for BMs treatment (Table 2). NcRNAs with more than 200 nucleotides are named as long ncRNAs (lncRNAs), while ncRNAs with less than 200 nucleotides are called small ncRNAs [91]. MicroRNAs are considered as small ncRNAs for regulating messenger RNA translation by binding to them and inhibiting protein production [92]. A series of microRNAs have been reported to play important roles for BM. MicroRNA-590 was found to downregulated in NSCLC cell lines with high metastatic potential and induced expression of microRNA-590 inhibited tumor cell proliferation, migration, and invasion [93]. Another study performed by Chen et al. uncovered that microRNA-375 was significantly downregulated in patients with BMs compared to NSCLC patients without BMs. Further analysis revealed that high level of microRNA-375 led to poor prognosis in patients through downregulation of matrix metalloproteinase-9 (MMP9) and VEGF [94]. Additionally, Singh et al. revealed that STAT3 knocking down could reduce lung cancer cell migration by its direct effect on microRNA-21 [95], suggesting STAT3 is a potential therapeutic target to prevent BMs.

**Table 2:** Summary of brain metastases-associated ncRNAs

ncRNAs	Type	Expression pattern in BMs	Related signaling pathways	Function for BMs	Reference
miR-590	microRNA	Downregulation in NSCLC-BM	Directly targeting ADAM9	Enhancing the migration and invasion of NSCLC cell lines with high metastatic potential	[81]
miR-375	microRNA	Downregulation in NSCLC-BM	Promoting the overexpression of VEGF and MMP9	Significantly shorten the OS of patients	[89]
miR-21	microRNA	Overexpression in LCBM	As a direct downstream target of STAT3 and binds to STAT3	Promoting migration and self-renewal of tumor stem cell populations, tumor cell proliferation, survival and migration	[90]
lnc-MMP2-2	lncRNA	Overexpression in NSCLC-BM	As a microRNA sponge or a competing endogenous RNA for miR-1207-5p	Increasing BBB permeability	[9]
*SLNCR*	lncRNA	Overexpression in melanoma-BM	Binding to Brn3a and AR to transcriptional activation of MMP9	Promoting invasion and leading to an overall lower survival	[91]
MALAT1	lncRNA	Overexpression in NSCLC-BM	Inducing EMT process	Promoting migration	[92]
RP11-355I22.7	lncRNA	Overexpression in BCBM	Increasing JAK2 kinase activity to mediate OSM- and IL-6-triggered STAT3 phosphorylation	Promoting BCBM by mediating communication between cancer cells and the brain microenvironment	[93]
BMOR	lncRNA	Overexpression in BCBM	Inactivating IRF3	Promoting BCBM by evading immune-mediated killing in the brain microenvironment	[94]
LGALS8-AS1	lncRNA	Overexpression in NSCLC-BM	Targeting miR-885-3p to mediate FSCN1 expression	Promoting cancer cell growth, angiogenesis	[95]

*ADAM9* a disintegrin and metalloproteinase 9, *AR* androgen receptor, *BBB* blood–brain barrier, *BCBM* breast cancer brain metastases, *BM* brain metastasis, *Brn3a* brain-specific homeobox protein 3a, *EMT* epithelial-mesenchymal transition, *FSCN1* fascin actin-bundling protein 1, *IRF3* interferon regulatory Factor 3, *JAK2* janus kinase-2, *LCBM* lung cancer brain metastasis, *lncRNA* long non-coding RNA, *miR* microRNA, *MMP9* matrix metalloproteinase 9, *ncRNA* non-coding RNA, *NSCLC* non-small cell lung cancer, *OSM* oncostatin M, *STAT3* signal transducers and activators of transcription 3

Similar to microRNAs, the lncRNAs also have been reported to play important roles for LCBM progression. High level of lncRNA-MMP2-2 increases BBB permeability and subsequently induces NSCLC metastases. Inhibition of lncRNA-MMP2-2 reduces BMs formation in vivo [9]. Another study performed by Schmidt et al. revealed that lncRNA-SLNCR1 interacted with androgen receptor and transcription factor Brn3a to form a complex to increase MMP9 expression and then induced melanoma cell invasion and migration [96]. LncRNA metastasis associated lung adenocarcinoma transcript 1 (MALAT1) was significantly upregulated in BMs than that in non-BM samples and the level of MALAT1 was associated with patients’ survival [97]. Increased level of MALAT1 promotes LCBM by inducing EMT, which may be a promising prognosis factor and therapeutic target to treat LCBM in future. Wang et al. have uncovered that lncRNA associated with BCBM (lnc-BM) was prognostic of the progression of BM in breast cancer patients [98]. Elevated lnc-BM expression drove BM and depletion of lnc-BM with nanoparticle encapsulated siRNAs effectively inhibited BM in preclinical mice model. Lnc-BM increased JAK2 activity to mediate oncostatin M and IL-6 triggered STAT3 phosphorylation to enhance BM. Liu et al. have revealed that a brain-enriched long noncoding RNA (BMOR) was expressed in breast cancer metastatic cells and was required for colonization of tumor cells in brain [99]. BMOR enables cancer cells to evade immune-mediated killing in the brain microenvironment by binding and inactivating interferon regulatory factor 3. Zhou et al. have revealed that NSCLC-BM patients exhibited higher level of LGALS8-AS1 than NSCLC patients without BMs [100]. Depleting LGALS8-AS1 prevented NSCLC cell proliferation, migration and angiogenesis in vitro and also inhibited NSCLC tumorigenesis and BM in vivo. LGALS8-AS1 may be a useful biomarker for identifying NSCLC with metastatic potential. Thus, the ncRNAs regulate BM formation and progression through different mechanisms and maybe served as potential therapeutic targets for BM treatment.

### Genomic and transcriptomic changes in lung cancer brain metastasis

Genomic sequencing of a large cohort of metastatic tumors and matched primary tumors has revealed previously unknown genomic aberrance of BMs. Skakodub et al. have uncovered that the tumor mutational burden, the fraction genome altered and the copy-number alterations were significantly higher in BM specimens compared to either primary tumor or extracranial metastases samples [80]. Additionally, genomic alterations of *CDKN2A/B*, *TP53*, *KRAS*, and *EGFR*, were more common in the BM samples compared to primary tumor samples. Using paired whole-exome and RNA-seq data, Martínez-Ruiz et al. have described the genomic-transcriptomic evolution in lung cancer and metastases [101]. They characterized the transcriptomes of primary-metastatic tumor pairs by combining multiple machine-learning approaches to link metastasis-seeding potential to the evolutionary context of mutations and increased proliferation within primary tumor regions. By performing WES of 40 samples from 12 LUAD-BM patients, Jiang et al. have observed significant higher intertumoral heterogeneity in BMs compared to pair-matched primary tumors [102]. Phylogenetic analysis revealed that BM-competent clones genetically diverged from their primary tumors at relatively early stage, suggesting a parallel progression model of genetic evolution trajectory. Saunus et al. have investigated the genomic and transcriptomic landscapes of 36 BMs from breast, lung, melanoma and esophageal cancers [103]. Using DNA copy-number analysis and exome- and RNA-sequencing, they identified novel candidates with possible roles in BM development, including the significantly mutated genes *DSC2, ST7, PIK3R1* and *SMC5*, and the DNA repair, ERBB-HER signalling, axon guidance and protein kinase-A signaling pathways. Treatment with tyrosine kinase inhibitors (TKIs) osimertinib that targeting activated EGFR can prolong the survival of patients with EGFR mutant lung cancer. However, patients often develop metastatic relapses to the brain. Using metastatic mouse models of EGFR mutant lung cancer, Biswas et al. have demonstrated that overexpression of S100A9 could escape osimertinib treatment and initiate BM [104]. S100A9 upregulates Aldehyde dehydrogenase 1A1 (ALDH1A1) expression and activates the retinoic acid (RA) signaling pathway in osimertinib-refractory cancer cells. Targeting S100A9-ALDH1A1-RA signaling could suppress BMs in EGFR mutant lung cancer patients. Wang et al. have performed WES and RNA-seq on 30 LUAD-BM patients and revealed that genomic drivers required for BMs often occur early in the primary lung tumor. In addition, they uncovered that genetic intratumor heterogeneity remodeled the immune microenvironment and induced immune evasion in LCBM [105]. Wei et al. have revealed that lysophosphatidylcholine acyltransferase 1 (LPCAT1) could promote LCBM by upregulating the PI3K/AKT/MYC pathway and maybe a promising target for treating LCBM patients [106]. Jiang et al. have silenced LPCAT1 using an exosome-based delivery system to efficiently arrest tumor growth and inhibit malignant progression of BM in vivo [107]. Thus, the genomic evolution and transcriptomic changes promote the transformation of primary lung cancer cells to more malignant forms for BMs and may provide potential therapeutic targets.

### Heterogeneity of lung cancer brain metastasis at single cell and spatial resolution

LCBM tumors have great intra- and intertumoral heterogeneity among patients. Despite aggressive treatment, the LCBM patients still exhibit dismal prognosis. Tumor heterogeneity, including malignant cell hierarchy and TME diversity, is assumed to responsible for the failure of treatment. Thus, comprehensively understanding the heterogeneity of LCBM tumors may provide the potential for improving therapeutic effects. The recent advantages of scRNA-seq technology have extended our insights into the molecular and cellular mechanisms of BM at single cell resolution (Table 3).

**Table 3:** Single-cell studies for lung cancer brain metastasis discussed in this review

Assay	Tissues profiled	Organism	Accession	Key findings	References
scRNA-seq	LUAD	Human	GSE131907 EGAD00001005054	LUAD-BM exists a special cancer cell subtype that dominates the late-stage and metastatic stage; normal myeloid cell populations of the metastasis site are gradually replaced with monocyte-derived macrophages.	[108]
scRNA-seq	LUAD	Human	GSE131907	S100A9 is specially expressed in a subgroup of BM original cells and its high expression level is positively associated with poor survival.	[109]
scRNA-seq	Lung cancer and other cancers	Human	GSE131907 GSE186344	Metastatic tumor cells were divided into eight subtypes and further identified two major categories: highly proliferative or highly inflammation.	[110]
CyTOF	NSCLC	Human	https://data.mendeley.com/datasets/jk8c3c3nmz/draft?a=c0a9d8dc-8ac2-4942-baf9-208de7a8c310	﻿Leukocyte invasion was higher in BM than that in brain resident tumours. BM tumors harbored a high frequency of regulatory T cells and monocyte-derived macrophages.	[111]
﻿Flow cytometry ﻿RNA-seq ﻿Protein arrays	Lung cancer and other cancers	Human	https://joycelab.shinyapps.io/braintime/	BM and IDH mutation types shape the brain TME; microglia and monocyte-derived macrophages exhibit multifaceted activation.	[112]
IMC	Lung cancer and other cancers	Human	GSE154795 GSE162631	BM tumors have increased frequency and density of NK cells, neutrophils, macrophages, classical monocytes, T cells and decreased dendritic cells and non-classical monocytes compared with glioblastoma,	[113]
scRNA-seq	Lung cancer and breast cancers	Human	GSE234832 GSE186344	LCBM components include tumor cells, fibroblasts, myeloid cells, stromal cells, oligodendrocytes and T cells. Tumor-associated fibroblasts secreting type I collagen are the key mediators of TME.	[114]

*scRNA-seq* single-cell RNA- sequencing, *LUAD* lung adenocarcinoma, *LCBM* lung cancer brain metastases, *NSCLC* non-small cell lung cancer, *CyTOF* cytometry by time-of-flight, *IMC* imaging mass cytometry, *TME* tumour microenvironment

#### Malignant heterogeneity and cellular origin of lung cancer brain metastasis

Recently, utilizing of scRNA-seq technology has revealed novel insights for the malignant heterogeneity and cellular origin of LCBM tumors, which has not been uncovered before using conventional technologies, such as bulk profiling. Kim et al. have performed single-cell transcriptome profiling of primary and metastatic LUAD and identified a special cancer cell subtype that deviated from normal differentiation trajectory and dominated the late-stage and metastatic stage [108]. Survival analysis revealed that this special cancer cell subtype associated gene sets could predict poor survival in LUAD patients. Furthermore, the normal resident myeloid cell populations were found gradually replaced with monocyte-derived macrophages and dendritic cells, along with T-cell exhaustion, which led to an immunosuppressive microenvironment. While endothelial cells and fibroblasts orchestrated tissue remodeling and angiogenesis to promote tumor progression and metastases. By analyzing the single-cell transcriptomic profiles of primary LUAD and LUAD-BM samples, Wang et al. demonstrated that LUAD-BM was derived from a subclones of primary LUAD with a gain of chromosome 7 and identified S100A9 as a biomarker of the cellular origins [109]. Expression level of S100A9 was strongly correlated with BM and prognosis. In addition, targeting these cellular origins by lapatinib could inhibit LUAD-BM. Gonzalez et al. have performed integrative analysis of single-cell transcriptomics and mass cytometry for malignant and non-malignant cells from 15 human BM tumors, including LCBM samples [110]. Eight functional cell programs that coexist or anticorrelated were delineated to two functional archetypes, one proliferative and the other inflammatory, which were shaped by tumor-immune interactions. Combining scRNA-seq and spatial transcriptomic analyses, Xiao et al. have identified a special early metastatic epithelial cell cluster (EMEC), which was enriched in OXPHOS and coagulation, was related to clinical prognosis [115]. In addition, EMEC cells invaded from the peripheral region to the central regions of the tumor with the progress of invasion. Thus, the heterogeneity and cellular origin of LCBM uncovered by scRNA-seq and spatial transcriptomics provide potential therapeutic targets for LCBM patients.

#### Heterogeneity of lung cancer brain metastasis microenvironment

Comprehensively defining the specific immunological signature of BM tumors can facilitate the rational design of immunotherapy strategies. Recently, two parallel studies performed by Friebel et al. and Klemm et al. have uncovered the distinct immunological microenvironment of BMs compared to brain resident tumors (Fig. 5b). By single-cell profiling analyses, Friebel et al. have revealed that leukocyte invasion was higher in BMs than that in brain resident tumors. BM tumors harbored a high frequency of regulatory T cells and monocyte-derived macrophages, whereas glioma was characterized by brain resident reactive microglia [111]. By high-dimensional multi-omics characterization of the brain TME, Klemm et al. found that BM types and glioma isocitrate dehydrogenase mutation status shaped the brain TME [112]. Microglia and monocyte-derived macrophages exhibited multifaceted activation in BM tumors and altering the multifaceted phenotypes of TAMs rather than simply depletion of these cells should be considered as an effective therapeutic strategy. Karimi et al. have applied imaging mass cytometry to characterize the single-cell spatial immune landscapes of high-grade glioma and BM tumors [113]. Compared to glioblastoma, increased frequency and density of NK cells, neutrophils, macrophages, classical monocytes, T cells and decreased in dendritic cells and non-classical monocytes were found in BM tumors. Additionally, increased monocytes and microglia were found in BM-cores of patients without leptomeningeal disease, suggesting a putative protective role for these cells. Furthermore, endothelial cells were found to have a high tendency to interact with Ki67-expressing tumor cells in both cores and margins of BM. However, within BM cores, rather than margins, tumor-adjacent endothelial cells exhibited lower claudin-5 expression compared with tumor-avoiding endothelial cells, supporting a model of vascular co-option during BM colonization. Song et al. have revealed the landscape of the human BM microenvironment by single-cell profiling and uncovered various intratumoral components, including tumor cells, fibroblasts, myeloid cells, stromal cells, oligodendrocytes and T cells [114]. Among them, type I collagen-secreting CAFs were identified as key mediators for remodeling the TME in BM tumors and associated with patient survival. By digital spatial transcriptomic profiling of lung tumors and metastases, Zhang et al. have provided a whole transcriptome map of LCBM resolved with morphological markers for the tumor core, tumor immune microenvironment, and tumor brain microenvironment [116]. They revealed that the brain TME was undergone extensive remodeling to create an immunosuppressive and fibrogenic niche for the BMs. Specifically, the brain TME is characterized with reduced antigen presentation, dysfunction of B cells and T cells, increased neutrophils and M2-type macrophages, immature microglia, and reactive astrocytes. Differential gene expression and network analysis identified fibrosis and immune regulation as the major functional modules disrupted in brain TME. In summary, the extensive single-cell and spatial analysis of TME of BM tumors enhances our understanding of the cellular dynamics and intercellular interactions and reveals the potential diagnostic and therapeutic targets.

## Therapeutic strategies

### Current treatment modalities

**Fig. 6:**
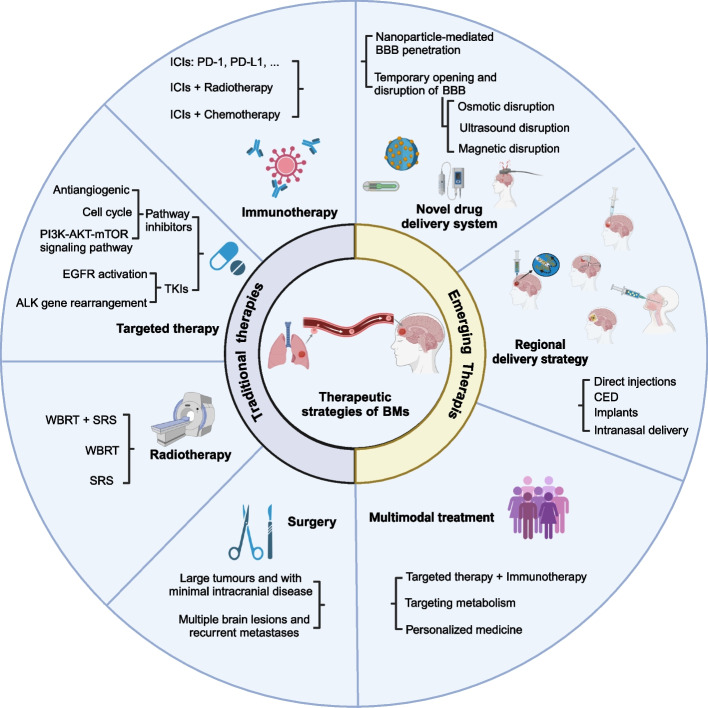
Current treatment strategies of BMs, including traditional and emerging therapy. The figure was generated using Biorender.com

Current treatment approaches of BM include surgical resection, whole-brain radiation therapy (WBRT), stereotactic radiosurgery (SRS), targeted therapy, immunotherapy or a combination of several modalities [13–15]. Since the intro- and inter-heterogeneity of BMs, there is no definitive consensus of the sequence of treatment and the outcomes are often volatile. Thus, the treatment for BM patients should be in a flexible way. Here, we summarized the current therapeutic strategies for BMs (Fig. 6).

#### Surgery, radiation therapy, and systemic treatments

Surgical resection should be considered for patients with large tumors and with minimal intracranial disease. BM patients usually have a better survival after surgical resection if they have a single and accessible BM lesion [117, 118]. For multiple brain lesions and recurrent metastases, the current advances in surgical techniques have further resulted in safer resections and promoted the benefit [118]. After surgical resection of both primary lesion and BM lesions, the long-term survival of LCBM patients is improved [119]. However, elevation of the serum carcinoembryonic antigen (CEA) level in NSLC-BM patients after surgical resection for solitary BM lesion may lead to postoperative recurrence. NSCLC-BM patients with normal CEA level, small primary tumor size, and node-negative status will be significantly benefit to local control and with improved survival rates after surgical resection of the primary tumor and synchronous BM [120]. A retrospective multicenter study proved the safety and importance of surgical treatment in NSCLC-BM patients without molecular driver alterations [121].

Patients with single or multiple BM lesions could be considered for WBRT or SRS strategy, which could also be considered for patients with karnofsky performance status < 70 or with comorbidities impeding neurosurgery. One study showed a median OS of 6 months treated with WBRT alone in 229 SCLC-BM patients [122]. Recently, it has been reported that WBRT was more recommended for NSCLC-MB patients with good status of performance and were unsuitable for SRS treatment, whereas not for patients with a poor prognosis [123]. Additionally, WBRT treatment has clear survival advantages for NSCLC patients with leptomeningeal metastasis and wildtype EGFR [124]. However, WBRT should be prudently used in the NSCLC-BM patients, due to therapeutic effect may be accompanied by worse cognitive decline [123].

As a safer and more effective technique, SRS has gradually replaced WBRT and represented a cornerstone of BM management. SRS exerts therapeutic effects by delivering high doses of radiation to tumor lesions, and its benefits include quick treatment, low rate of complications and less deterioration of neurocognitive functions [125]. It has been reported that SCLC-BM patients treated with upfront SRS exhibited favorable outcomes, including 1-year control of 80% and median survival time of approximately 8 months [126]. A meta-analysis has also demonstrated that upfront SRS produced favorable lesion control and survival outcomes when used for SCLC-BM patients with limited BM lesions [127]. Small retrospective case series have indicated that 189 NSCLC-BM patients with fewer than 4 BM lesions and less than 4 cm in size had longer survival after treated with SRS alone than those treated with WBRT [128]. In oligometastatic NSCLC-BM patients, treatment for both lung primary lesion and BM lesions with lung synchronous thoracic stereotactic body radiation therapy and brain SRS achieved good local control rates and led to improved OS [129]. Previous studies suggested that SRS could be as a promising salvage treatment after WBRT for SCLC-BM patients [130, 131]. Andrews et al. reported significant better survival for combination of WBRT with SRS group in single BM patients compared to WBRT only group [132]. However, given the combination of SRS with WBRT is associated with potential adverse effects, such as higher rate of cognitive impairment, hippocampal avoidance or administration of memantine is necessary to decrease neurological toxicity.

Surgical resection and radiotherapy are the major local treatment approaches for BM patients currently. However, the advanced cancer patients usually cannot benefit from local therapies as multiple tumor lesions in the brain and other parts of the body. On the other hand, patients with asymptomatic BM have increased over the last decade. Recent clinical trials for systemic treatment, such as immune and targeted therapies, exhibited promising intracranial responses especially in asymptomatic status. Current available systemic therapies for BM patients include chemotherapy, targeted therapy, endocrine therapy, immunotherapy, and novel therapies that may be available in the future. Systemic treatments are often used to complement local strategies to achieve optimal control of BM [133]. With the emergence of various new targetable drugs, the systematic therapies have shown promising clinical results recently. Several next-generation TKIs and immune checkpoint inhibitors have shown significant intracranial efficacies. Osimertinib and afatinib have exhibited remarkable intracranial response rates for asymptomatic NSCLC-BM patients. Immune-related therapy and next-generation TKIs have been applied for in advanced cancer patients with BM in the last decades. However, systemic therapies may come with some side effects, patients need to be closely monitored and manage these side effects during the treatment process.

#### Challenges in drug delivery across the blood–brain barrier

Although systemic therapies exhibit promising effects for BM treatment, the chemotherapy and targeted therapy are challenged for administering in BM patients’ treatment because of the BBB. The BBB is composed of endothelium cells, vascular basement membrane, pericytes, and astrocyte endfeets [134, 135]. Endothelial cells form the impermeable BBB by surrounding astrocytes and the basement membrane. BBB can separate circulating blood and brain tissue. The BBB is both a physiological and biochemical obstacle that protects the brain from potentially hazardous chemicals while also maintains the brain homeostasis [136]. However, the impermeable property of BBB also limits the transport of therapeutic agents to the brain, making systemic therapies less effective in treating BMs than in treating other types of cancers. As BMs formed in the brain, the BBB is disrupted and replaced by BTB [137]. Although the structure is damaged to a certain degree, BTB can impede the transport of anticancer agents to brain tumors, leading to poor treatment effect. Since the BBB and BTB are impediments for drug delivery to brain, disruption techniques are needed to improve the permeability of drugs to metastatic tumors in the brain. Invasive and noninvasive techniques for improving antitumor agent delivery across the BBB and BTB are being developed. Recent advances have confirmed the systemic delivery of therapeutic agents via transporter systems, nanoparticle-based systems, molecular trojan horse technology, and engineered stem cells, which are promising approaches for treating BMs. Identifying the molecular mechanisms and signaling pathways underlying BBB and BTB formation may lead to the discovery of new strategies for BMs treatment.

### Targeted therapies

Driver mutation identifications have greatly promoted the targeted therapies for lung cancer and LCBM (Table 1). Most targeted therapies for LCBM are those targeting driver mutations of *EGFR*, *ALK*, *ROS1*, *RET*, and *MET*, as well as these driver mutation-associated signaling pathways, such as RAS/RAF, PI3K/AKT/mTOR, WNT/β-catenin, and JAK/STAT, which also provide new strategies for the treatment of LCBM [79, 80, 82–84, 86, 88, 138]. In light of this, there are ongoing targetable clinical trials enrolling patients based on the molecular characteristics [85, 87, 139–148].

#### Inhibitors of key molecules

Genetic and genomic analyses have identified driver mutations for the development of LCBM. Among these drivers, mutations in *EGFR* activation and *ALK* gene rearrangement are of particularly important owing to the high prevalence in BM patients and the clinical availability of targeted inhibitors [149–152]. LCBM is heterogeneous and associated with specific driver oncogenes. Around 15 ~ 20% of non-squamous NSCLC patients have *EGFR* mutations at diagnosis [153]. In clinical practice of TKIs treatment, erlotinib, the first-generation EGFR-TKI, is the first-line systemic option for metastatic patients with activated *EGFR* mutations and exhibits significant response for LCBM with objective response rate (ORR) of 51.8% [140]. Afatinib is the second-generation EGFR-TKI that has been shown to improve progression free survival (PFS) of *EGFR*-mutated NSCLC-BM patients with ORR of 70 ~ 75% and PFS of 8.2 months [85]. The data from a retrospective study indicated that patients with *EGFR* mutations treated with TKIs gefitinib or afatinib have better outcomes [87]. However, both the first and second generation EGFR-TKIs finally resulted in acquired treatment resistance [154]. Recently, it has been reported that the third-generation EGFR-TKIs osimertinib could overcome the most common resistance and exhibited more effective BBB penetration than previous generation TKIs in preclinical and early clinical studies with ORR of 40 ~ 70% and PFS of 18.9 months [141, 155].

*ALK* rearrangements were detected in around 5% of newly diagnosed patients with non-squamous NSCLC [153]. ALK inhibitors are small molecule targeted drugs and the clinical trials of first-generation ALK-TKI crizotinib showed a better disease control rate of 18 ~ 62% and extended PFS and OS with the median intracranial time to progression is 7 ~ 13.2 months [142, 143]. The second-generation ALK-TKIs alectinib (brain response rate, 52.4%) and ceritinib (brain response rate, 42.1%) were used to treat NSCLC-BM patients with *ALK* active mutation and crizotinib resistance. However, these inhibitors exhibited adverse CNS effect [143]. Brigatinib (ORR, 42 ~ 67%; median PFS, 9.2 ~ 12.9 months) is a third-generation ALK-TKI and was applied to crizotinib-resistant NSCLC-BM patients [144, 156]. Another third generation ALK inhibitor lorlatinib is still in the phase 3 CROWN study, which can improve PFS (median duration of follow-up for PFS, 36.7 months), ORR (76 ~ 82%), duration of response (≥ 2 years, 74%), and time to intracranial progression (≥ 2 years, 81%) of advanced NSCLC patients [157]. In presence of asymptomatic BMs, deeper CNS ORR and better brain penetrance were observed for upfront treatment with second (alectinib) or third (brigatinib and lorlatinib) generation ALK inhibitors compared to crizotinib [151, 158, 159]. Among these TKIs, lorlatinib exhibited the highest cerebral spinal fluid (CSF)-plasma ratio and preserved intra-cranial activity compared to alectinib and brigatinib, with a CNS ORR of 59% and PFS of 24.6 months [145, 160]. Several other targetable driver mutations were identified in NSCLC-BM, including *NTRK*, *RET*, and *neuregulin 1* (*NRG1*) [161]. Among these targetable driver mutations, TKI is the main therapy strategy for targeting *NTRK* and *RET* active mutations. A clinical trial indicated that a novel bispecific HER2/3 antibody (MCLA-128) led to intracranial response in NSCLC-BM patients with NRG1 fusion [162].

#### Pathway inhibitors

﻿In addition to genomic driver mutations, molecular analyses have identified signaling pathways that dysregulated and associated with BMs. A topic of clinical interest is whether these pathways offer effective therapeutic targets for BMs treatment. Bohn et al. have indicated that combinatorial inhibition of VEGF and Angiopoietin-2 (Ang2) decreased lesion permeability and reduced BMs of breast cancer burden in animal model [163]. Another study performed by Kovalchuk et al. also have revealed that VEGF pathway inhibitor nintedanib and dual anti‑VEGF/Ang2 nanobody selectively prevented BM of LUAD cells [164]. Both compounds were able to normalize cerebral blood vessels within BMs lesions, indicating a brain specific effect of antiangiogenic compounds BI836880 with respect to metastasis treatment. Nintedanib and BI836880 are promising candidates for future BM preventive study concepts in LUAD patients. Brastianos et al. have presented a clinical trial for cyclin-dependent kinase (CDK) inhibitor by a small cohort of BM patients with CDK alterations and met the primary endpoint [165]. They evaluated the intracranial efficacy of CDK inhibition and demonstrated a 53% intracranial benefit rate at 8 weeks with palbociclib in a pretreated population of patients with progressive BM and CDK alterations. Aberrant activation of the PI3K-AKT-mTOR signaling pathway is frequently observed in many cancers, including primary tumors and BMs [166]. Agents targeting key components of this pathway have demonstrated antitumor activity in diverse cancers and may represent a new treatment strategy for BMs. Several inhibitors of this pathway have been demonstrated to penetrate the BBB and down-regulate PI3K signaling in preclinical studies, indicating that these agents may be potential therapies for BMs. The PI3K inhibitor buparlisib and the mTOR inhibitor everolimus are currently under evaluation in combination with trastuzumab in patients with HER2^+^ breast cancer BMs.

### Immunotherapies and their challenges in the brain metastases context

Recent clinical guidelines agree that NSCLC patients with asymptomatic BMs and without oncogenic drivers should be treated with immune checkpoint inhibitors (ICIs) [139]. The usage of ICIs is an attractive strategy to stimulate immune response of the immunosuppressed TME [57, 167]. ICIs are now commonly used in the management of NSCLC patients, particularly for patients without molecularly targetable mutations. The ICIs targeting programmed death receptor 1 (PD-1) (pembrolizumab and nivolumab) and PD-L1 (atezolizumab) as well as cytotoxic T lymphocyte-associated antigen-4 (CTLA4) (ipilimumab) have been developed and evaluated in LCBM patients [168]. Treatment recommendations for tumors with a PD-L1 expression 0 ~ 49% include platinum-based chemotherapy with anti–PD-1/PD-L1. Tumors with a PD-L1 expression ≥ 50% benefit from the prior mentioned regimens or anti-PD-1/PD-L1 agents in monotherapy. Clinical trials have demonstrated that patients with asymptomatic BM benefited from the nivolumab-ipilimumab combination, reporting an intracranial benefit of 57%, and an OS at 3 years of 71.9% [169–171]. A multicenter retrospective analysis including 100 NSCLC-BM patients treated with upfront ICIs showed a CNS ORR of 27% among evaluable patients and rates of 35.7% in PD-L1-positive and 11.1% in PD-L1-negative tumors [172].

Since NSCLC-BM patients treated with ICIs alone usually exhibited low-to-modest response, combination of ICIs with radiotherapy or/and chemotherapy is needed to be further investigated [173, 174]. A meta-analysis of 19 studies in NSCLC patients reported that combination of ICIs and radiation was associated with prolonged OS and with no significant increase in neurologic adverse events in comparison with radiation alone [175]. Other studies also indicated that PD-1 or PD-L1 antibody alone or in combination with chemotherapy or radiotherapy exhibited activity for both NSCLC- and SCLC-BM patients [176, 177]. A series of prospective trials with the regimens of testing combinations of ICIs and SRS are ongoing or in development. Chang et al. have demonstrated that immunotherapy improved the OS and intracranial PFS of SCLC-BM patients [178]. At least four cycles of ICIs should be applied for SCLC patients with BMs and cross-line treatment with ICIs is recommended. Given the low survival rates of SCLC patients, the use of ICIs should be as early as possible. They also showed that immunotherapy plus chemotherapy and radiotherapy for BMs exhibited promising efficacy for SCLC-BM patients.

With the widespread application of immunotherapy, a series of challenges had emerged during the treatment process. One of the issues is the penetration and efficiency of activation of immune cells for the immune checkpoint inhibitors. Another issue that requires resolution is that of pseudo-progression, a potentially fatal intense inflammatory response that can mimic rapid tumor progression [179]. Although the optimal diagnosis and management strategies for pseudo-progression remain active areas of study, immune depression with a course of steroids is often pursued. Systemic toxicity with immunotherapies is also a concern, with mild toxicity rates as high as 50%, although rates of grade 3 or 4 toxicities were less than 10%. To overcome the challenges of immunotherapies, a better understanding of the regulatory mechanisms underlying immune infiltration and activation is necessary, which will lead to improve the incorporation of immunotherapies into the therapeutic landscape for BMs.

## Emerging therapies and future directions

### Novel drug delivery systems

#### Nanoparticles and other blood–brain barrier-penetrating strategies

Cancer therapy has been remarkably improved over the past few years because of the development of antitumor agents at the nanoscale. More recently, biomaterial-based nanodrugs have been developed and applied in preclinical studies and clinical trials of cancer immunotherapies [180, 181]. Nanoparticles used in targeted drug delivery systems exhibit unique advantages, such as reliable delivery, sufficient loading capacity, nanosized, and drug-loading plasticity [182]. Nanodrugs have increased the efficacy and reduced the toxicity of parent drugs in clinical trials of BMs treatment [183]. Nanoparticle-mediated targeted delivery of chemotherapeutic drugs to BMs is currently among the most frequently used approaches. BBB can prevent macromolecules and nanoparticles from shuttling freely between the blood system and BM tumors. However, the interactions between nanoparticles and BBB, including biological interactions and physicochemical interactions, can be utilized to delivery of nanoparticles to BM tumors. The biological interactions include receptor-mediated transcytosis (RMT) and transporter-mediated transcytosis (TMT), while the physicochemical interactions contain absorption-mediated transcytosis (AMT) and BBB opening.

RMT is considered as the most promising method and is widely used for delivery of nanoparticles with the characterization of high specificity, selectivity, and affinity for brain tumors [180, 181]. Most endogenous macromolecules are transported into brain parenchyma via RMT. RMT-based transport is usually energy-dependent and with relatively high efficiency. To achieve a best delivery efficiency of therapeutic to a brain tumor, the target receptor should be highly expressed in the endothelial cells of the brain tumor, whereas minimally expressed in other vascular endothelial cells to minimize the off-target effect. The typical receptors utilized in nanoparticle-based drug delivery for brain tumors includes transferrin receptor mediated transcytosis, low-density lipoprotein receptor-mediated transcytosis, insulin receptor-mediated transcytosis and nicotinic acetylcholine receptor-mediated transcytosis. TMT is also known as carrier-mediated transcytosis, which is a vital strategy for transporting low molecular weight nutrients from the bloodstream into the brain [180, 184]. Many transporters have been discovered on the BBB. Among them, glucose transporter and glutathione transporter are the two most explored transporters for facilitating nanoparticles crossing the BBB in brain tumor treatment. AMT is another widely explored approach for delivering drugs across the BBB. AMT is triggered by the electrostatic interaction between positively charged agents and negatively charged luminal membranes of brain endothelial cells [181]. Cationic bovine serum albumin and cell-penetration peptides are two kinds of the most frequently used moieties conjugated on nanoparticles to trigger nanoparticles across the BBB through AMT [185]. Notably, although the travel route of AMT is similar to RMT, there is a significant difference between these two pathways. The prominent feature of AMT is its high binding capacity to BBB cells and associated with poor tissue selectivity due to non-special binging.

Temporary opening and disruption of BBB by using biochemical reagents or physical approaches is another straightforward and effective strategy for agent brain delivery [186, 187]. Different from interaction mediated nanoparticle transport, which is nearly not influence the integrity of BBB, the disruption of BBB by physicochemical method usually compromises the integrity of BBB to a certain degree temporarily and reversibly. Therapeutic agents then transport into the brain parenchyma by diffusion after temporary opening the BBB. The commonly adopted disruption methods include hyperosmotic agents induced osmotic disruption, ultrasound disruption, magnetic disruption, and their orthogonal combination.

#### Regional delivery strategies

Besides the nanoparticle delivery, regional drug delivery strategies, such as direct injections, convection enhanced delivery (CED), administration of implants, and intranasal delivery (IN), have been developed to bypass the BBB and enhance the therapeutic efficacy of treatment agents. Although these strategies are invasive, site-specific delivery of the therapeutics with high bioavailability and minimal drug loss can be achieved. Drugs can be directly injected or infused into the brain tumor sites. This strategy is less toxic and much effective than systemic administration. However, this method exhibits high risk of side effects such as edema and infection [188].

CED is developed to prevent the backflow of drugs and improve the drug distribution in brain tumor site [189]. In this method, the catheter is introduced stereotactically while the constant pressure from the pump maintains a convection flow of the drug solution into the delivery site. In addition, the convective flow allows the drug solution to cover longer distance in the brain compared to direct injection/infusion [190]. CED has been widely tested for the delivery of a broad spectrum of therapeutic agents, including small molecules, macromolecules, nanocarriers, and immunotoxins [191–195]. Despite the significant promise, invasiveness and the common side effects of infusion methods are limitations for CED strategies. Polymeric implants, including wafers, gels, microspheres and nanospheres, have been tested for regional delivery of therapeutic agents in brain tissue. Wafers are drug loaded polymeric implants and are implanted in the remaining cavity of post-surgical resection of a tumor where they act as a platform for sustained drug release [196]. The major drawback of wafers as standard delivery platform for treating invasive brain tumors is lack of deep tissue penetration. Unlike wafers and other large implants, gels and microsphere- and nanosphere-based or polymeric microchips have been tested as alternate drug delivery implants. However, due to the common limitation of lack of deep tissue penetration, none of these are clinically approved [188]. IN is another non-invasive approach to circumvent the BBB. Although the mechanism of intranasal drug delivery to brain is not well understood, the olfactory and trigeminal pathway is presumed to be the significant route for nose-to-brain drug delivery [197]. Some clinical trials of IN for the brain disease treatment are ongoing.

### Combination therapies and multimodal treatments

#### Synergistic approaches combining targeted therapies with immunotherapies

Targeted therapies can be more effective and less harmful than traditional chemotherapy or radiotherapy [198]. However, cancer cells can activate alternative signaling pathways to bypass the blocked pathways targeted by special inhibitors and gain an adaptive resistance. Immunotherapies activate the body’s immune cells against cancer cells and represent the most promising strategy for cancer therapy. However, cancer cells also become resistant to immunotherapies through the downregulation of antigens that immune cells recognize, making themselves less visible to the immune system [199]. In addition, the presence of immunosuppressive TME also contributes to the low efficacy of immunotherapies [200, 201]. To address the adaptation of cancer cells to targeted therapy and resistance to immunotherapy, scientists have explored combination strategies that targeted therapies combined with immunotherapies based on the complementary mechanisms of action and potential for synergistic interaction [202, 203].

Small-molecule targeted therapies have been reported to exert specific effects on antitumor immune response in mouse models and in the clinical trials. Inhibitors of v-Raf murine sarcoma viral oncogene homolog B (BRAF), CDK4/6 and poly ADP-ribose polymerase 1/2 (PARP1/2) are currently being tested in combination with immune checkpoint blockades (ICBs) in clinical trials and have shown promising potential. Treatment with BRAF inhibitors has been shown to increase NK-cell infiltration and reduce Treg and MDSC levels in mouse models of BRAF-mutant melanoma [204–206]. Furthermore, BRAF inhibition with dabrafenib in combination with the MEK inhibitor trametinib also enhanced CD8^+^ T-cell infiltration and improved response to PD-1 blockade in animal model [207]. In vitro small-molecule screen has identified CDK4/6 inhibitors as capable of directly enhancing T-cell activation via NFAT signaling, which was required for proper activation and function of T cells [208]. CDK4/6 inhibition by palbociclib or trilaciclib has been reported to potentiate PD-1 blockade to stimulate antitumor T-cell function and inhibit tumor growth in preclinical studies. In vivo treatment with CDK4/6 inhibitors increased PD-L1 levels of tumor cells and sensitized ICBs, resulting in tumor regression in mice receiving combined palbociclib and anti-PD-1. It has been reported that the therapeutic efficacy of PARP inhibitors (PARPis) required not only the direct cytotoxicity, but also the coordinated activation of robust local and systemic antitumor immune response [209, 210]. PARPis have also been shown to induce expression of PD-L1 in tumor cells [211–214], which could promote the adaptative immune suppression. Preclinical studies have shown that PD-1/PD-L1 blockade further augmented PARPi-triggered immune response, leading to more durable suppression of tumor growth and prolonged survival.

Recent studies have revealed that PD‑L1 and VEGF dual blockade enhanced anti‑tumor effect on BMs in animal model. The combination of anti-PD-L1 antibody and anti-VEGF antibody showed a stronger anti-tumor effect than each single agent [215]. Anti-PD-L1 antibody alone enhanced CD8^+^ T cell priming in regional lymph nodes and increased the density of CD8^+^ cells in the brain tissue. Anti-VEGF antibody alone decreased microvessel density in BM lesions. PD-L1 blockade combined with VEGF blockade increased the antitumor effect by increasing the infiltration of activated CD8^+^ T cell and decreasing microvessel density. LCBM have posed a significant clinical challenge due to acquired resistance to TKI treatment. Fu et al. have revealed that TKI treatment elevated the immune checkpoint CTLA4 expression in T cells, promoting an immune-suppressive microenvironment [216]. Combination of CTLA4 blockade with TKI treatment enhanced efficacy over TKI monotherapy, highlighting the potential of CTLA4 blockade in effectively overcoming TKI resistance in LCBM.

#### Metabolic reprogramming as a therapeutic target

Metabolic alterations observed in BMs provides potential targets for new therapeutics. Since cancer cells have higher nutrient consumption than normal cells, targeting metabolism represents a potential therapeutic perspective. The enzymes that participate in biochemical reactions during metabolism provide many target-specific targets that can be exploited pharmacologically. Given that normal brain cells use the same metabolic pathways as BMs, it is important to account for the potential off-target toxicities of metabolic-targeted therapeutics. Some metabolic interventions have already exhibited minimal toxicity profiles. 

Ferraro et al. have revealed that fatty acid synthesis was required for breast cancer BMs [217]. Fatty acid synthesis was elevated in BCBM, which was an adaptation to decreased lipid availability in the brain relative to other tissues. Genetic or pharmacological inhibition of fatty acid synthase reduces breast tumor growth in the brain, demonstrating that differences in nutrient availability across metastatic sites can result in targetable metabolic dependencies. Furthermore, both primary brain tumors and metastatic tumors show upregulated glycolysis and OXPHOS for which many potential drugs, including metformin and phenformin, that target bioenergetic pathways have been identified as therapeutic alternatives in the clinical setting [218–221]. The treatment of cancer cells with dichloroacetate, an inhibitor of mitochondrial pyruvate dehydrogenase kinase, led to normalize glucose oxidation, making the cancer cells susceptible to apoptosis while normal cells are unaffected [222]. Dichloroacetate is also well tolerated in mice and in humans [223, 224]. These studies demonstrate the potential benefit of targeting metabolic liability and flexibility to treat BMs. On the other hand, inhibition of key metabolic factors in brain tumor cells is challenging, as neurons and glial cells are metabolically active, and their inhibition can lead to CNS toxicity. Furthermore, many drugs fail to penetrate brain tissue due to the presence of formidable BBB, making the design of BM-targeting therapies very challenging. Effective drug delivery methods combined with metabolic targets may provide advances in the treatment of BMs.

### Personalized medicine and precision medicine

#### Biomarker-driven treatment strategies

Biomarker testing is recommended for patients diagnosed with NSCLC and already implemented in diagnostic and therapeutic routine workups. Patients with targetable mutations are associated with a higher incidence of BMs, but also with a prolonged survival prognosis, particularly after the introduction to targeted therapy [225]. Approximately 15 ~ 20% of non-squamous NSCLC patients present with an EGFR mutation and routine testing of EGFR mutation is recommended in metastatic NSCLC with non-squamous histology [226, 227]. EGFR mutations predict response to therapy with TKIs. Specifically, the third-generation EGFR-TKI osimertinib presented remarkable intracranial response rates of over 80% in patients with EGFR-mutated metastatic lung cancer and asymptomatic BMs.

The ALK rearrangements occur in 2 ~ 9% of LUAD patients [228]. Therefore, ALK testing is recommended in patients with non-squamous histology if EGFR mutations were not detected [229]. ALK rearrangements predict response to ALK-TKIs. The next-generation ALK inhibitors, including alectinib, lorlatinib, and brigatinib, were associated with considerable intracranial response rates over 65% [230–232]. Importantly, ALK-TKIs are further effective in BM patients with ROS1 rearrangement. ROS1 gene rearrangements are present in approximately 1 ~ 2% of non-squamous NSCLC patients and serve as a predictive biomarker for response to ALK-TKIs [233]. Considering the remarkable intracranial response rates in ROS1-altered LUAD patients treated with TKIs, regularly testing for ROS1 in clinical setting should be evaluated [234]. BRAF V600E mutation is detected in 2 ~ 4% of NSCLC patients, therefore, routine testing for this biomarker in BMs from NSCLC are currently not generally recommended. However, recent phase II clinical trials have postulated the clinical efficacy of BRAF inhibitor dabrafenib in combination with the MAPK kinase inhibitor trametinib as a therapeutic approach in metastatic BRAF V600E-mutated NSCLC. Moreover, considering the success of dual inhibition in BRAF V600E-mutated melanoma BMs, further clinical trials focusing on the intracranial efficacy of dabrafenib and trametinib in BRAF-mutated NSCLC BM are warranted.

#### Patient-derived xenograft (PDX) models for personalized therapy

Establishment of a disease model that adequately reflects the characteristics of BM tumors is crucial to the development of novel therapeutics. PDX models have a high clinical relevance, offering the potential for individualized patient treatment. Previous research demonstrated that successful PDX modeling can be achieved by direct intracranial implantation of metastatic cells or tissue into the lateral ventricle and frontal cortex. Orthotopic intracranial implants allow cancer cells to more closely resemble their original patient tumors both phenotypically and genotypically given the influence of the brain microenvironment.

Antitumor activity against BMs can be assessed in PDX models, which may be especially relevant when implanted orthotopically into the brain to more closely reproduce the anatomic situation of the original tumor. Baschnagel et al. have reported on the development and characterization of a cohort of NSCLC-BM PDX models, including both flank and orthotopic intracranial xenografts [235]. These PDXs models have been assessed their response to radiation and selumetinib in *KRAS* mutated xenografts and savolitinib in a *MET* exon 14 mutated xenograft. Friese-Hamim et al. have investigated the brain penetration and efficacy of tepotinib in orthotopic PDX models of *MET*-driven NSCLC-BM [236]. Guillen et al. have established a human breast cancer-derived xenograft and organoid platform for drug discovery and precision oncology [237]. They leveraged PDXs and PDX-derived organoids for drug screening and demonstrated the feasibility of using these models for precision oncology in real time with clinical care in a case of triple-negative breast cancer. A FDA-approved drug eribulin with high efficacy against the models was identified and treatment with this therapy resulted in a complete response for the individual.

 Faria et al. have established a library of 23 PDXs of BMs from eight primary tumors that resembled the disseminated disease of the patients [238]. These PDXs and their matched BMs have similar gene expression profiles and recapitulate the dissemination pattern of patient tumors. These PDXs were tested in preclinical studies for anticancer therapies, such as the FDA-approved drugs of pan-PI3K inhibitor buparlisib and mTOR inhibitor everolimus. Sun et al. have made comprehensive characterization of 536 PDX models and prioritized candidates for targeted treatment [239]. These studies uncovered that development of candidate cancer treatments was a resource-intensive process, with the research community continuing to investigate options beyond static genomic characterization. Compared with human tumors, PDXs typically have higher purity and fit to investigate dynamic driver events and molecular properties via multiple time points from same case PDXs. These PDX models of BMs recapitulate the biology of human metastatic disease and provide valuable methods and resources for functional precision medicine and drug development for human BMs.

## Conclusion

BM is the leading cause of death in lung cancer patients with 40% of NSCLC patients and 40–50% of SCLC patients develop BMs during their disease course, leading to high morbidity and mortality rates. Despite the advances of therapeutic strategies in the past decades, the prognosis is still grim and the molecular and cellular mechanisms of BMs have not been fully elucidated. Current LCBM trials predominantly target NSCLC and tyrosine kinase inhibitors emerged as promising treatments for brain parenchymal and leptomeningeal NSCLC metastases (Table 4). For example, a phase II trial of atezolizumab combined with carboplatin and pemetrexed showed activity in advanced and untreated NSCLC-BM patients with an acceptable safety profile [240]. BM is a complex process involves a series of pathological stages, which can be divided into three major stages according to the location of incident: primary lung cancer stage, CTCs stage, and BM stage. Each step of the BM process is tightly regulated by distinct molecular and cellular mechanisms. However, most of the current studies focused on the third stage of the interaction between tumours cells and brain microenvironment. Due to the lack of animal models, the intrinsic signature of dissociated cancer cells and molecular changes before entering brain are usually less studied. Thus, a deeper understanding of the BM process for both before and after entering the brain may help to develop more and effective potential therapeutic strategies.

**Table 4:** Clinical trials directed towards brain metastases in recent years

Identifier	Phase	BM Type	Patients included	Treatment	Started year	Status
NCT03297788	II	SCLC	SCLC patients with BM	SRS or WBRT	2017	Completed
NCT04824079	II	NSCLC	Advanced NSCLC patients with BM after treatment with EGFR inhibitors	Keynatinib	2020	Recruiting
NCT05012254	II	NSCLC	Stage IV or recurrent, NSCLC patients with synchronous BM	Treatment with two cycles of Platinum-based chemotherapy (Carboplatin or Cisplatin) plus Nivolumab and Ipilimumab; then maintain with Nivolumab and Ipilimumab	2021	Active, not recruiting
NCT04631029	I	SCLC	Extensive stage SCLC patients with BM	Entinostat in combination with Atezolizumab/Carboplatin/Etoposide	2021	Completed
NCT05104281	III	NSCLC	NSCLC patients with BM and *EGFR* mutation	Osimertinib combined with Bevacizumab	2021	Recruiting
NCT06128148	I	NSCLC	NSCLC patients with BM and *ROS1* fusion	JYP0322 (an orally available inhibitor of ROS1)	2022	Recruiting
NCT04967417	II	NSCLC	Stage IV NSCLC patients with BM	Pemetrexed, Carboplatin and Pembrolizumab or Paclitaxel, Carboplatin and Pembrolizumab	2022	Recruiting
NCT05948813	II	NSCLC	Patients with EGFR-mutated NSCLC with BM	TY-9591; Osimertinib	2023	Recruiting
NCT06238882	not applicable	NSCLC	Patients with stage IV NSCLC with *EGFR* mutations and BM	Total CRT plus concomitant transdermal nitroglycerin	2023	Recruiting
NCT06476093	not applicable	NSCLC	NSCLC patients with BM resistant to treatment	SRT combined with Anlotinib (a novel TKI)	2024	Recruiting
NCT06501391	II	NSCLC	Stage IV NSCLC patients with BM and without driver gene mutations	PD-L1/PD-1 inhibitor and chemotherapy combined with SRT or WBRT	2024	Recruiting
NCT06676917	II	NSCLC	Non-squamous NSCLC with BM	Datopotamab Deruxtecan (Dato-DXd; a tumor-associated calcium signal transducer 2 (TROP2)-directed antibody drug conjugate)	2025	Not yet recruiting
NCT03696030	I	BC	HER2 positive BC patients with BM	Autologous HER2-targeted chimeric antigen receptor (HER2-CAR) T cells	2018	Recruiting
NCT05781633	II	BC	BC patients with BM	A regimen of eutidrone, etoposide and bevacizumab	2022	Recruiting
NCT06152822	II	BC	HER2 positive BC patients with BM	Pyrotinib combined with capecitabine and bevacizumab	2023	Recruiting
NCT05872347	II	BC	HR-positive, HER2-negative BC patients with BM	SPH4336 (a novel highly selective oral CDK4/6 inhibitor)	2023	Recruiting
NCT06088056	II	BC	HER2 positive BC patients with BM	SRT combined with Trastuzumab-Deruxtecan (T-DXd)	2023	Not yet recruiting
NCT06418594	II	BC	HER2-negative BC patients with BM	Adebrelimab plus apatinib and etoposide	2024	Recruiting
NCT06462079	II	BC	HER2-negative BC patients with BM	Sacituzumab Govitecan combined with intracranial radiotherapy	2024	Not yet recruiting
NCT06210438	II	BC	Triple-negative BC patients with BM	SHR-A1921 (an antibody conjugated drug targeting Trop-2) combined with bevacizumab	2024	Not yet recruiting
NCT03563729	II	Melanoma	Melanoma patients with BM and in need of steroid treatment	Pembrolizumab alone or ipilimumab and nivolumab in combination	2018	Recruiting
NCT03898908	II	Melanoma	Patients with BRAF mutant melanoma metastatic to the brain	Encorafenib and binimetinib	2019	Active, not recruiting
NCT04074096	II	Melanoma	BRAFV600 mutation-positive melanoma patients with BM	Adding upfront SRS to binimetinib-encorafenib-pembrolizumab combination therapy	2022	Active, not recruiting
NCT05704933	I	Melanoma	Melanoma patients with BM	A single dose of nivolumab with ipilimumab or relatlimab	2023	Active, not recruiting
NCT06712927	II	Melanoma	Melanoma patients with BM	Relatlimab, nivolumab, and ipilimumab	2025	Not yet recruiting

All information comes from https://classic.clinicaltrials.gov

*BC* breast cancer, *CRT* cranial radiation therapy, *EGFR* Epidermal Growth Factor Receptor, *SRT* stereotactic radiotherapy, *SRS* stereotactic radiotherapy, *WBRT* whole brain radiation therapy

Over the past decades, advancements in cancer therapeutics have markedly improved patient outcomes, resulting in longer survival and subsequently a potential rise in the incidence of BMs. Although novel therapies such as immunotherapies and targeted therapies exhibit promising results in controlling extracranial disease, the efficacy of systemic therapies for intracranial metastases is still grim and treatment options for BMs remain limited. This is partly attributed to the special structure of the brain, especially the obstruction of BBB that can impede the antitumor agents delivery into brain. To solve this issue, biomaterial-based nanodrugs have been developed and applied in preclinical studies and clinical trials of BMs. Nanoparticles used in targeted drug delivery systems exhibited unique advantages and have increased the efficacy and reduced the toxicity of parent drugs in clinical trials of BMs treatment.

Despite the advances in treatment have significantly increased life span of BM patients, the incidence of BMs has been steadily increasing and the prognosis for BM patients remains poor. In addition, the current therapeutic strategies often lead to serious adverse. BM patients are often accompanied with extracranial metastases, which must be addressed along with BMs. Since the tumoral heterogeneity of BM patients, the treatment strategies for BM patients should be in a flexible way. Therefore, fully understanding the molecular and cellular mechanisms of BMs, as well as the development of new effective therapeutic interventions based on the newly identified targets, will ultimately change the dismal prognosis of BM patients.

## Data Availability

Not applicable.

## Abbreviations

ABL: Abelson
ALDH1A1: Aldehyde dehydrogenase 1A1
ALK: Anaplastic-lymphoma-kinase
AMT: Absorption-mediated transcytosis
Ang2: Angiopoietin-2
BBB: Blood–brain barrier
BM: Brain metastases
BMDMs: Bone-marrow-derived myeloid cells
BMOR: Brain-enriched long noncoding RNA
BRAF: V-Raf murine sarcoma viral oncogene homolog B
CAFs: Cancer-associated fibroblasts
cCAFs: Circulated CAFs
CDK: Cyclin-dependent kinase
Cd-pericytes: Cell-derived pericyte-like cells
CEA: Carcinoembryonic antigen
CED: Convection enhanced delivery
cGAMP: 2′3′-cyclic GMP-AMP
CITE-seq: cellular indexing of transcriptomes and epitopes by sequencing
CTCs: Circulating tumour cells
CTLA4: Cytotoxic T lymphocyte-associated antigen-4
CyTOF: cytometry by time-of-flight
DCCs: Disseminated cancer cells
DHA: Docosahexaenoic acid
ECM: Extracellular matrix
EGFR: Epidermal growth factor receptor
EMEC: Early metastatic epithelial cell cluster
EMT: Epithelial-mesenchymal transition
ICBs: Immune checkpoint blockades
ICIs: Immune checkpoint inhibitors
IL-6: Interleukin 6
IN: Intranasal delivery
L1CAM: L1 cell adhesion molecule
LCBM: Lung cancer brain metastasis
lnc-BM: LncRNA associated with BCBM
lncRNAs: Long ncRNAs
LND: Lonidamine
LPCAT1: Lysophosphatidylcholine acyltransferase 1
LUAD: Lung adenocarcinoma
MALAT1: Metastasis associated lung adenocarcinoma transcript 1
MAPK: Mitogen-activated protein kinase
MET: Mesenchymal-to-epithelial transition
Mfsd2a: Major facilitator superfamily domain 2a
Mito-LND: Mito-lonidamine
MMP9: Matrix metalloproteinase-9
MSLN: Mesothelin
ncRNAs: Non-coding RNAs
NK cell: Natural killer cell
NMDARs: N-methyl-d-aspartate receptors
NRG1: Neuregulin 1
NSCLC: Non-small cell lung cancer
NTRK: Neurotrophic tropomyosin receptor kinase
ORR: Objective response rate
OS: Overall survival
OXPHOS: Oxidative phosphorylation
PA: Plasminogen activator
PARP 1/2: Poly ADP-ribose polymerase 1/2
PARPis: PARP inhibitors
PD-1: Programmed death receptor 1
PDGF: Platelet-derived growth factor
PD-L1: Programmed death ligand 1
PFS: Progression free survival
PHGDH: Phosphoglycerate Dehydrogenase
PLGF: Placental growth factor
RA: Retinoic acid; RET: rearranged during transfection
RMT: Receptor-mediated transcytosis
ROS: Reactive oxygen species
ROS1: ROS proto-oncogene 1
SCLC: Small cell lung cancer
scRNA-seq: Single-cell RNA- sequencing
SRS: Stereotactic radiosurgery
STING: stimulator of interferon genes
TAM: Tumor associated macrophages
TEM: Trans-endothelial migration
TKIs: Tyrosine kinase inhibitors
TME: Tumour microenvironment
TMT: Transporter-mediated transcytosis
ULBP: UL16-binding proteins
VEGFA: Vascular endothelial growth factor A
WBCs: White blood cells
WBRT: Whole-brain radiation therapy
